# Gut microbiota in chronic inflammation: the interplay with lipid mediators

**DOI:** 10.1080/19490976.2026.2667605

**Published:** 2026-05-06

**Authors:** Suxia Bao, Chengcan Yao

**Affiliations:** a Centre for Inflammation Research, Institute for Regeneration and Repair, The University of Edinburgh, Edinburgh, UK; b Department of Infectious Disease, Shanghai Ninth People's Hospital, Shanghai Jiao Tong University School of Medicine, Shanghai, China

**Keywords:** Gut microbiota, dysbiosis, microbial metabolites, microbial products, dietary fatty acids, lipid mediators, eicosanoids, chronic inflammation, inflammatory bowel disease, metabolic diseases, arthritis

## Abstract

The gut microbiota plays a fundamental role in maintaining host health by regulating immune function, epithelial barrier integrity, and metabolic homeostasis. Disruption of microbial community structure (also known as dysbiosis) and altered host–microbiota interactions can shift microbial composition and metabolite production, promote immune dysregulation, and contribute to the initiation and persistence of chronic inflammation. Eicosanoids, a class of signaling lipid mediators derived from arachidonic acid , are essential modulators of acute and chronic inflammatory responses. Emerging evidence highlights a bidirectional interplay between the microbiota and eicosanoid pathways as a hallmark of chronic inflammation. Microbial taxa and their metabolites regulate arachidonic acid availability, eicosanoid biosynthesis, and receptor signaling in host cells. In turn, host-derived eicosanoids shape the gut environment, influencing the gut microbiota and host health state. This self-reinforcing loop drives key features of chronic inflammatory diseases, including a shift toward pro-inflammatory eicosanoid profiles, a relative deficiency of anti-inflammatory or pro-resolving lipid mediators, and microbiota dysbiosis. In this review, we summarize recent advances in the mechanisms underpinning microbiota-eicosanoid crosstalk, outline its contribution to chronic inflammatory diseases, and discuss the therapeutic potential of targeting this bidirectional axis.

## 1 Introduction

The gut microbiota forms a vast and complex ecosystem of microorganisms that resides within the human gastrointestinal tract. It plays a fundamental role in aiding digestion, regulating immune function, maintaining gut epithelial homeostasis, and supporting whole-body health.^1-5^ In this context, an imbalance in the microbiota, known as dysbiosis, including an increase in the abundance of harmful taxa and a reduction of beneficial taxa, is associated with impaired gut barrier integrity and systemic inflammation, driving the development and progression of various diseases. Gut microbiota dysbiosis can profoundly influence disease induction through the altered production of microbial molecules and metabolites.^3^ Mechanistically, dysbiosis often leads to an increase in pro-inflammatory bacterial components such as lipopolysaccharide (LPS), which can translocate across a compromised intestinal barrier, often termed “leaky gut”, and trigger systemic inflammation.^6^^,^^7^ Additionally, imbalances in microbial metabolites can disrupt host metabolic and immune homeostasis.^8^ These alterations collectively contribute to the onset and progression of metabolic, inflammatory, and autoimmune diseases. Thus, microbial-derived metabolites serve as critical mediators linking gut dysbiosis to host pathophysiology.

**Figure 1.**
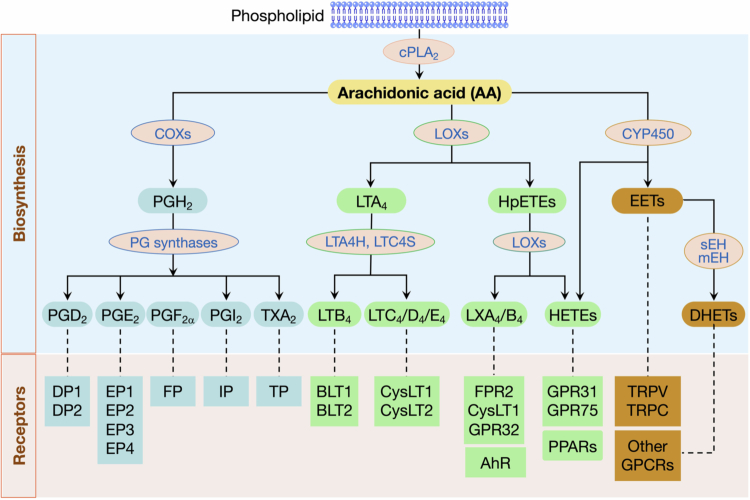
Eicosanoid biosynthesis and their receptors. Arachidonic acid (AA) is released from membrane phospholipids by cytosolic phospholipase A_2_ (cPLA_2_) and is subsequently oxidized by cyclooxygenases (COXs), lipoxygenases (LOXs), and cytochrome P450 (CYP450) enzymes, respectively, to generate intermediate products. These intermediates are then converted into eicosanoids, including prostaglandins (PGs, e.g., PGD_2_, PGE_2_, PGF_2α_, and PGI_2_), thromboxane A_2_ (TXA_2_), leukotrienes (e.g., LTB_4_ and LTC_4_), lipoxins (e.g., LXA_4_), hydroxyeicosatetraenoic acids (HETEs), epoxyeicosatrienoic acids (EETs), and DHETs, by specific terminal synthases, such as prostaglandin synthases, LTA_4_ hydrolase (LTA4H), LTC_4_ synthase (LTC4S), lipoxygenases (LOXs), and soluble epoxide hydrolase (sEH), respectively. Bioactive eicosanoids signal primarily via cognate cell surface G protein-coupled receptors, although some can also act through nuclear receptors such as the aryl hydrocarbon receptor (AhR) and peroxisome proliferator-activated receptors (PPARs).

Chronic inflammation often arises from persistent, low-grade immune activation, which can be triggered by metabolic stress, microbial dysbiosis, and tissue damage.^9-11^ These inflammatory programs are shaped by downstream effector systems, such as the key eicosanoid networks.^12^^,^^13^ Eicosanoids are bioactive lipid mediators derived from polyunsaturated fatty acids (PUFAs) such as arachidonic acid (AA). Their biosynthetic pathways and major signaling receptors are outlined in Figure 1. In the gut, they regulate immune responses, epithelial barrier function, and tissue remodeling. Their biological effects are shaped by (i) the spectrum of eicosanoids generated by distinct enzymatic pathways, (ii) the receptor landscape of local target cells, (iii) the state of tissue (homeostasis versus disease), which positions them as key mediators of microbiota-host inflammatory crosstalk, and (iv) the timing of lipid signaling across immune activation and tissue injury, inflammation, and repair. In this context, microbial dysbiosis and eicosanoid dysregulation can amplify each other, creating a self-perpetuating loop to sustain inflammation. Shifts in the gut microbial community can directly and indirectly influence host eicosanoid pathways. For instance, gut microbiota can metabolize dietary fats, including PUFAs, oxidizing them into precursors of lipid mediators and thereby reprogramming the balance of eicosanoids.^14^^,^^15^ Reciprocally, changes in the host eicosanoid signaling can modify the intestinal microenvironment, which in turn reshapes microbiota community structure and function.^16^^,^^17^ Collectively, microbiota–host eicosanoid crosstalk represents a critical mechanism that drives the pathogenesis of chronic inflammatory diseases, acting both locally within the gut and at systemic sites.

In this review, we examine bidirectional crosstalk between the gut microbiota and eicosanoid pathways in the context of chronic inflammation. We summarize current evidence for how microbiota community shifts contribute to eicosanoid imbalance and, in turn, how dysregulated eicosanoid signaling reshapes the microbiota composition and function, collectively influencing host homeostasis and the development of chronic inflammatory diseases. We then detail the specific roles of these pathways in a range of chronic inflammatory diseases, including inflammatory bowel disease (IBD), metabolic disorders, and arthritis. Furthermore, we examine emerging evidence supporting the therapeutic potential of targeting these pathways for disease modulation.

## 2 Microbiota regulation of host eicosanoid pathways

**Figure 2.**
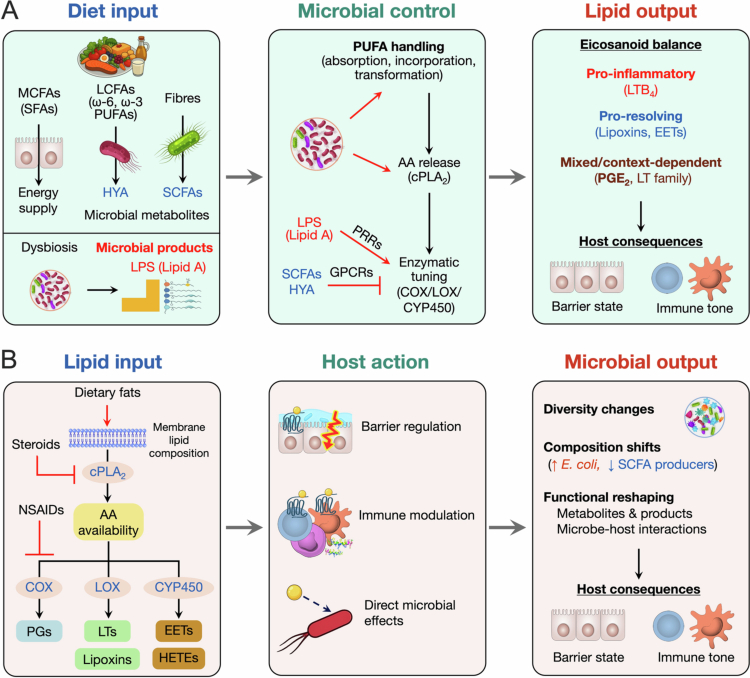
Microbiota-eicosanoid directional interactions. (A) Diet-derived microbial metabolites (e.g., SCFAs, HYA) and dysbiosis-associated microbial products (e.g., LPS and related Lipid A) influence PUFA handling, cPLA_2_-mediated AA mobilization, and the activity of key eicosanoid-biosynthetic enzymes (COX, LOX, CYP450). These signals reprogramme intestinal eicosanoid profiles, shaping epithelial barrier function, immune responses, and inflammatory outcomes. (B) Dietary fatty acids influence membrane lipid composition and the supply of substrates for cPLA2-mediated release of arachidonic acid (AA). Steroids suppress cPLA_2_ activity, whereas NSAIDs inhibit COX-dependent PG biosynthesis and can reciprocally enhance LOX- and CYP450-mediated eicosanoid pathways. The resulting host-derived eicosanoids regulate the gut epithelial barrier niche, shape mucosal immune programs, and in some instances, affect microbial growth. Together, these actions alter microbiota diversity and metabolic output, with consequences for intestinal and systemic health.

The gut microbiota plays a critical role in shaping host lipid mediator profiles by controlling both substrate availability and the host enzymatic programs that mobilize these substrates and generate bioactive mediators.^18-20^ Microbial community members and their metabolites influence the intestinal handling of dietary components, including fibres, medium-chain fatty acids (MCFAs) and long-chain fatty acids (LCFAs, including PUFAs), by affecting their absorption and incorporation into cell membrane phospholipid pools, and by modulating phospholipase-dependent release of AA, a major precursor for host eicosanoids. In parallel, the microbiota can also regulate lipid mediator metabolism by tuning eicosanoid synthase expression and activity, for example via activation of pattern recognition receptors (PRRs) on host cells (Figure 2A). Together, these mechanisms reprogram the intestinal eicosanoid profile and provide a mechanistic link between microbial dysbiosis and chronic inflammatory conditions.

### 2.1 Microbial shaping of eicosanoid precursor pool

Upon entering the digestive tract, dietary fats are predominantly hydrolyzed and absorbed by host cells (primarily enterocytes) in the small intestine, supplying LCFAs that are incorporated into cellular membrane lipid pools. While the gut microbiota has a limited direct role in LCFA digestion, it can aid the metabolism of unabsorbed lipids and shape lipid uptake by influencing host cell emulsification processes.^21^ LCFAs comprise saturated fatty acids (SFAs), monounsaturated fatty acids (MUFAs), and PUFAs, such as AA, eicosapentaenoic acid (EPA), and docosahexaenoic acid (DHA).

PUFAs are key structural components of cellular membranes and serve as precursors for bioactive lipid mediators, such as eicosanoids. The eicosanoid precursors are primarily obtained from dietary fat intake, with AA arising from dietary linoleic acid (*ω*-6 PUFA) and EPA/DHA from *α*-linolenic acid (*ω*-3 PUFAs). Following intake, these fatty acids are incorporated into the host cell membrane, released into the cellular plasma by the actions of phospholipases, and then converted into downstream lipid mediators.^22^ Dietary *ω*-6 versus *ω*-3 PUFA balance can bias the overall eicosanoid profile. While high *ω*-6 PUFA intake favors AA-derived pro-inflammatory mediators, *ω*-3 PUFA supplementation generally increases anti-inflammatory and/or pro-resolving eicosanoids.^23^^,^^24^

The microbiota can further skew the process by biotransforming dietary PUFAs, thereby shifting the availability and balance of different eicosanoid precursors.^21^ For example, gut microbes can directly generate oxylipins from dietary EPA and DHA.^14^ Notably, *Lactobacillus species* convert dietary linoleic acid into 10-hydroxy-cis-12-octadecenoic acid (HYA), which limits the pool of AA available for proinflammatory eicosanoid synthesis.^25^ Furthermore, gut microbes directly metabolize PUFA-derived epoxides into diols, shifting the colonic eicosanoid profile by reducing colonic levels of epoxide-type eicosanoids and increasing downstream diol metabolites.^20^ Beyond these specific biotransformations, the gut microbiota can also convert some dietary PUFAs into conjugated and more saturated products via isomerization and hydrogenation,^26^ lipolysis of complex lipids, and actively convert linoleic acid into bioactive intermediates, thus increasing the availability of precursors and intermediates for downstream eicosanoid production.^27^^,^^28^

Although the gut microbiota is not generally considered a direct source of LCFAs, certain bacterial strains can be enriched in specific LCFA species. For example, *Escherichia coli* Nissle 1917 contains higher levels of the anti-inflammatory LCFA 3-hydroxyoctadecaenoic acid (C18-3OH) than other *E. coli* strains.^29^ More broadly, the gut microbiota can significantly promote intestinal LCFA utilization and absorption,^30^ improve intestinal lipid sensing,^31^ and facilitate lipid storage and metabolism,^32^ therefore shaping the substrate landscape for host lipid mediator generation.

### 2.2 Microbiota control of eicosanoid precursor mobilization

Besides substrate availability, eicosanoid output also depends on the mobilization of membrane fatty acids, particularly AA release driven by phospholipase activation. The microbiota can prime and potentiate phospholipase activity to drive the release of AA from membrane phospholipids and subsequently expand the substrate pool availability for eicosanoid biosynthesis.^20^^,^^22^^,^^33^ In inflammatory settings, microbial dysbiosis further promotes AA release by triggering pro-inflammatory factors that activate the innate sensing pathway. Collectively, microbiota-dependent control of phospholipase activation provides a critical mechanistic link between dysbiosis and AA monilisation, thereby enabling tuning of downstream eicosanoid biosynthetic enzymes and pathways.

### 2.3 Microbiota regulation of eicosanoid biosynthesis

Once AA is released, eicosanoid biosynthesis proceeds through three major enzymatic branches (Figure 1). The cyclooxygenase (COX) pathway generates prostaglandins (PGs) and thromboxane via downstream PG synthases.^12^^,^^13^ The lipoxygenase (LOX) pathway produces leukotrienes (LTs), lipoxins, and some hydroxyeicosatetraenoic acids (HETEs), with key downstream enzymes including LTA_4_ hydrolase (LTAH) and LTC_4_ synthase (LTC4S). The cytochrome P450 (CYP450) pathway generates additional HETEs and epoxyeicosatrienoic acids (EETs), which are further converted to DHETs by soluble and microsomal epoxide hydrolases (sEH and mEH).^20^ COX and LOX enzymes can also oxidize *ω*-3 PUFAs such as DHA and EPA, further expanding the spectrum of bioactive lipid mediators. The final mediator profile is dictated by cell type, anatomical niche, and inflammatory context, which together determine the expression and activity of terminal synthases that convert intermediates into distinct eicosanoid species. Consistent with pathway-specific regulation, microbiota-dependent metabolites can activate CYP450-mediated protective eicosanoids (e.g., DHETs and EETs), with reported hepatoprotective effects in liver injury and fibrosis models.^34^

#### 2.3.1 Innate sensing pathways control eicosanoid enzymes

Microbiota-driven regulation of eicosanoid biosynthesis is frequently initiated by recognition of microbial components and stimulation by microbial metabolites. PRR activation by bacterial molecules can upregulate key eicosanoid-generating enzymes, including cytosolic phospholipase A_2_ (cPLA_2_) and downstream COX and LOX enzymes. Mononuclear phagocytes (macrophages, monocytes, and dendritic cells) are major intestinal sources of eicosanoids and respond robustly to bacterial and fungal stimulation by inducing cPLA_2_ and COX-2, subsequently increasing eicosanoid output.^16^^,^^35^ Consistently, *Clostridium difficile* infection induces COX-2 overexpression in human colonocytes via toxin A-induced reactive oxygen species and p38 MAPK signaling, whereas the probiotic *Lactobacillus rhamnosus GG* increases COX-2 expression in colonic myofibroblasts through TLR4-MyD88 signaling.^36^^,^^37^ Infections can trigger an “eicosanoid storm” characterized by COX-2 and 5-LOX upregulation and excessive production of inflammatory eicosanoids such as PGE_2_, PGD_2_, and cysteinyl leukotrienes.^13^^,^^38^ *Staphylococcus aureus* infection similarly increases COX-2- and mPGES1-mediated inflammatory eicosanoids while repressing 15-LOX-mediated specialized pro-resolving mediators in macrophages.^39^ By contrast, probiotic *Lactobacillus* and *Bifidobacterium* reduce COX-2 expression and pro-inflammatory eicosanoid production (e.g., PGE_2_) in macrophages.^40^

#### 2.3.2 Microbial metabolites tune eicosanoid enzymes

Microbiota-derived metabolites fine-tune eicosanoid enzyme expression and activity. Among the best-characterized microbial metabolites are short-chain fatty acids (SCFAs), C2–C5 fatty acids primarily generated from the fermentation of dietary fiber. Major SCFAs (acetic, propionic, butyric, and valeric acid) exert diverse physiological effects on immune regulation, metabolic control, tissue regeneration, and cancer biology.^41^ In the gut, SCFAs promote the induction of intestinal regulatory T cells,^42-44^ stimulate the release of enteroendocrine hormones,^45^ and support intestinal epithelial integrity.^46^

SCFAs also directly modulate eicosanoid synthases, but their effects are cell type- and context-dependent. In subepithelial myofibroblasts, SCFAs shift prostaglandin profiles by enhancing PGE_1_ while reducing PGE_2_. Through the greater potency of PGE_1_, this promotes epithelial MUC2 expression and reinforces mucosal barrier integrity.^47^ In human mononuclear cells (including monocytes), SCFAs, particularly *n*-butyrate, can synergize with TLR agonists to upregulate COX-2 and, via a G protein-coupled receptor-dependent mechanism, amplify PGE_2_ secretion.^48^^,^^49^ Conversely, SFCAs can epigenetically suppress key eicosanoid-synthesizing enzymes (including COX-2 and mPTGES) through histone deacetylase inhibition in the contexts of colorectal carcinogenesis and vascular smooth muscle cell function.^50^^,^^51^ In addition, receptor-dependent SCFA signaling (e.g., via GPR43 and GPR41) in enterocytes can promote intestinal inflammatory responses in chemical- or infection-induced colitis models.^52^ Beyond these direct effects, SCFAs can indirectly reshape mucosal immune tone and thereby alter the gut microbiota composition, creating feedback loops that ultimately influence the host intestinal eicosanoid landscape.

Beyond SCFAs, the microbiota intersects with other fatty acid classes that can influence the intestinal inflammatory milieu and microbial ecology. Medium-chain fatty acids (MCFAs), including caproic, caprylic, capric, and lauric acid, are mainly derived from dietary medium-chain triglycerides and are absorbed by intestinal epithelial cells. Although MCFAs are not direct eicosanoid precursors, they can indirectly influence lipid mediator outputs by modulating epithelial integrity and immune tone. MCFAs support energy expenditure and lipid oxidation in various tissues such as the liver and brain^53-55^ and promote epithelial renewal, barrier repair, and reduced mucosal inflammation.^56^^,^^57^ They can also exert antimicrobial activity that reshapes microbiota community structure by reducing pathogenic loads and potentially contribute to host resistance to infection.^58^^,^^59^ MCFA levels are reduced in patients with IBD,^60^ although immune associations can be context-dependent, for example, caproic acid has been positively associated with inflammatory interferon (IFN)-*γ*-producing CD4^+^ type 1 T helper (Th1) cells in multiple sclerosis.^61^ Age-associated microbiota shifts, especially expansion of *Parabacteroides goldsteinii,* has also been linked to increased MCFA production (notably 3-hydroxyoctanoic acid), engaging GPR84-expressing peripheral myeloid cells, impairing vagal afferent interoceptive signaling, and thereby reducing hippocampal activation and memory in aged mice.^62^

### 2.4 Dysbiosis-driven pathophysiological eicosanoid skewing

During dysbiosis, changes in microbial product composition can amplify innate sensing pathways, reinforce AA mobilization, and promote eicosanoid-dependent inflammatory milieu. Dysbiosis contributes to chronic inflammatory conditions such as IBD, metabolic diseases, and arthritis. In IBD, dysbiosis is often marked by expansion of *Proteobacteria*, a feature associated with epithelial stress and inflammation.^63-65^ Beyond taxonomic changes, dysbiosis can increase the inflammatory potency of microbial products. For example, it can drive remodeling of lipid A, the bioactive portion of LPS, through enhanced activity of bacterial modifying enzymes (e.g., *PagP*, *LpxR*, and *LpxP*), augmenting acylation and reducing phosphorylation levels, and then enhancing lipid A affinity for the toll-like receptor 4 (TLR4)-MD-2 complex.^66^^,^^67^ Functionally, hypo-acylated lipid A antagonizes TLR4-mediated pro-inflammatory interleukin (IL)-8 production in Crohn's disease (CD), whereas hyper-acylated lipid A activates TLR4 to potentiate IL-8 secretion in ulcerative colitis (UC).^68^ Therefore, lipid A remodeling represents a key mechanism linking microbial imbalance to pro-inflammatory eicosanoid signaling. In turn, TLR4 engagement activates MyD88- and TRIF-dependent MAPK signaling that phosphorylates and activates cPLA_2_ and COX-2, facilitating AA release and subsequent eicosanoid production.^69^ Collectively, dysbiosis-driven rewiring of microbial immunogenicity sustains AA mobilization and heightens eicosanoid output during inflammation.

Overall, the gut microbiota modulates host lipid mediator metabolism and signaling through complementary mechanisms that act on eicosanoid precursor pools (PUFAs), precursor mobilization (AA release), and enzymatic programming (COX/LOX/CYP pathways and terminal enzymes, e.g., PTGES, PTGDS, PTGIS, PTGFS, TBXAS), with additional contributions from metabolite signaling and local environmental cues. By reshaping the pool of eicosanoid precursors, dietary fat composition and microbial lipid handling influence downstream eicosanoid biosynthesis. Concurrently, microbiota-derived metabolites (e.g., SCFAs) and microbial products (e.g., LPS) differently tune key enzymatic checkpoints that determine precursor availability and/or eicosanoid biosynthetic processes, recalibrating eicosanoid output and balance. Together, these mechanisms provide a high-level framework for microbiota-driven reprogramming of lipid mediator pathways across homeostatic and inflammatory settings in a context-dependent manner.

## 3 Eicosanoid regulation of the gut microbiota

Eicosanoids exert their biological effects mainly through cell membrane G protein-coupled receptors, with distinct eicosanoid classes engaging different receptor families and downstream signaling pathways. For example, prostaglandin E_2_ (PGE_2_) signals via EP1–EP4 receptors, leukotrienes (e.g., LTB_4_, LTC_4_) bind to BLT1/2 and CysLT1/2 receptors, and lipoxins (e.g., LXA_4_) activate FPR2 (formyl peptide receptor 2) and other receptors (Figure 1). In addition, eicosanoids, their precursors, and downstream metabolites can signal through nuclear receptors. Lipoxins and CYP450-generated eicosanoids are ligands for the arylhydrocarbon receptor (AHR),^70^^,^^71^ while several oxidized eicosanoids (e.g., 15d-PGJ_2_, 15-keto-PGE_2_) and HETEs can activate peroxisome proliferator-activated receptors (PPARs).^72^^,^^73^ Through these receptor- and context-dependent pathways, eicosanoids can reshape the gut microbiota by altering gut epithelial barrier and mucus niches, modulating mucosal immune responses and antimicrobial programs, and in some cases directly influencing microbial growth. Upstream dietary and pharmacological interventions further perturb this axis by changing lipid input and membrane composition, AA availability and mobilization, and COXL/LOX/CYP450 pathway output, with downstream consequences for microbial diversity, composition, and function (Figure 2B).

### 3.1 Eicosanoid–immune interaction reshaping of the microbiota

#### 3.1.1 PGE_2_-driven microbiota shifts

PGE_2_ is a key regulator of intestinal homeostasis, supporting immune function, epithelial integrity, and containment of bacterial translocation, primarily through EP4 signaling in epithelial cells, innate lymphoid cells, and macrophages.^74^^,^^75^ Emerging evidence also indicates that PGE_2_-EP4 signaling can feed back onto the microbiota, but the direction and functional consequences of these effects are highly context dependent. At steady state, PGE_2_-EP4 signaling in myeloid cells reshapes the gut microbiota by reducing SCFA-producing bacteria and dampening myeloid interferon production, which in turn lowers colonic regulatory T cells (Tregs), especially RORγt-expressing Tregs with enhanced regulatory capacity.^17^ In contrast, during T cell-mediated intestinal inflammation, myeloid-specific EP4 signaling facilitates a dysbiotic shift characterized by expansion of pro-inflammatory taxa (notably Proteobacteria, especially *Escherichia*) and loss of segmented filamentous bacteria (SFB) adhesion to the epithelium. This microbial dysbiosis amplifies pathogenic T cell responses and exacerbates inflammation.^16^ Together, these studies illustrate that the same eicosanoid receptor pathway can drive microbiota outcomes depending on tissue state.

Consistent with eicosanoid-mediated control of microbial ecology, the PGE_1_ analog misoprostol leaves baseline microbiota largely unchanged but restores microbial diversity and community structure after antibiotic perturbation. In particular, misoprostol enriches beneficial taxa such as *Porphyromonadaceae* and *Akkermansia* while reducing *Bacteroides* overgrowth, thereby reducing tissue damage and protecting mice against *Clostridioides difficile* infection.^76^

#### 3.1.2 Impact of the intestinal barrier

By modulating key physiological functions, eicosanoids also maintain intestinal barrier integrity and limit translocation of bacteria and their products, therefore altering microbial persistence and community stability. Through EP4, PGE_2_ stimulates epithelial cell mucin secretion and enhances epithelial junctional protein expression by promoting IL-22 production from group 3 innate lymphoid cells (ILC3s).^77^ However, eicosanoid effects on the barrier can be context-dependent and may become disruptive under inflammatory conditions. In epithelial models, PGE_2_ increases intracellular calcium and activates myosin light chain kinase through EP1- and EP4-associated PLC-IP_3_ and cAMP-IP_3_-Ca^2+^ signaling, thereby redistributing tight junction proteins and increasing paracellular permeability.^78^ In parallel, inflammation-associated fibroblasts can disrupt epithelial architecture through paracrine PGE_2_-EP4 signaling that activates a PKA-CFTR axis, driving transepithelial fluid secretion and epithelial cell dysfunction.^79^ Furthermore, inflammatory stimuli [such as tumor necrosis factor (TNF)-*α*] increase the expression of PG transporters in epithelial cells, promoting vectorial transport of PGE_2_ (generated by myeloid cells in the lamina propria) from the basolateral (interstitial) compartment to the apical (luminal) surface. Because EP4 is typically expressed on the apical surface of epithelial cells, the resulting rise in luminal PGE_2_ preferentially activates apical EP4 signaling, ultimately disrupting colonic epithelial barrier integrity.^80^ Together, these mechanisms indicate that eicosanoids can either reinforce or undermine epithelial niches, regulating microbial access to the mucosa and shaping the microbial community.

#### 3.1.3 Direct effects on microbes

Eicosanoids may also influence microbes more directly, although the underlying mechanisms remain incompletely defined and require further investigation. For example, PGE_2_ has been reported to promote *E. coli* growth through direct and indirect host-mediated mechanisms, including immunosuppression of lymphocyte activity. Consistent with this, elevated PGE_2_ levels correlate with increased bacterial colonization and disease progression.^81^ Conversely, PGE_2_ has also been reported to synergize with antibiotics to enhance killing of *Staphylococcus aureus.*^82^ However, as there is currently no evidence that *S. aureus* synthesizes PGE_2_ or expresses cognate PGE_2_ receptors, the mechanistic basis of this observation remains unclear.

### 3.2 Reshaping microbiota via perturbations of eicosanoid tone

#### 3.2.1 Impact of dietary fats and eicosanoid precursors

Dietary fats, including eicosanoid precursors, can reshape gut microbiota diversity and composition with important consequences for intestinal inflammation. Supplementation with 20:4 *ω*-6 AA, which increases the dietary *ω*-6/ω-3 ratio, is associated with increased abundance of the *Escherichia-Shigella* genus and reduced *Bifidobacterium pseudolongum* in the colonic microbiota.^83^ These microbial shifts coincide with altered eicosanoid profiles and increased expression of inflammatory genes, including IL-1β and CD40.^83^

By contrast, supplementation with *ω*-3 PUFAs such as EPA and DHA generally enhances microbial diversity and enriches beneficial genera such as *Veillonella* and *Dialister.*^84^ Multiple studies further report a shift toward SCFA-producing taxa, including *Lactobacillus, Bifidobacterium, and Akkermansia*, with accompanying increases in SCFA production.^85^^,^^86^ EPA supplementation has also been reported to reduce LPS-producing bacteria within Bacteroidetes while increasing butyrate-producing Firmicutes, and to enrich beneficial taxa (e.g., *Akkermansia muciniphila* and Bacteroidetes), increase SCFA production, and reduce Proteobacteria.^87^^,^^88^ Similarly, dicosapentaenoic acid increases microbial diversity and selectively enriches genera such as *Akkermansia*, *Alistipes*, *Butyricicoccus*, and *Lactobacillus*, consistent with enhanced production of anti-inflammatory and barrier-protective metabolites.^89^

#### 3.2.2 Impact of pharmacological modulation of eicosanoid biosynthesis

Non-steroidal anti-inflammatory drugs (NSAIDs), which inhibit COX enzymes and thereby reduce prostaglandin synthesis, are associated with disruption of the gut microbial community and NSAID enteropathy. Indomethacin, a non-selective COX inhibitor, significantly alters small intestinal microbiota composition and community structure in association with epithelial injury.^17^^,^^77^^,^^90^^,^^91^ A time-course study further showed disruption of the mucosal biofilm, indicating broader changes in microbiota architecture.^92^ NSAID-associated microbiota changes, including enrichment of *Bacteroides*, *Akkermansia*, and *Parasutterella* and reduction of *Turicibacter*, can contribute to exacerbation of *Clostridioides difficile* infection and related intestinal inflammation.^93^ Another NSAID, Ketorolac, similarly increases small intestinal Proteobacteria despite limited effects on overt intestinal mucosal damage.^94^ In humans, aspirin, ibuprofen, and celecoxib have also been reported to significantly shape the gut microbiota, although effects on specific bacterial taxa vary across drugs.^95^ Use of standard dose of aspirin (325 mg) has been suggested to increase the relative abundance of taxa linked to SCFA-associated community structure, including *Akkermansia, Ruminococcaceae* and *Prevotella* and relatively decrease *Parabacteroides, Bacteroides* and *Dorea* in healthy volunteers.^96^ Overall, while direct NSAID-microbe interactions remain incompletely resolved, pharmacological perturbations of eicosanoid pathways can reshape microbial communities largely through host-mediated effects on barrier and immune tone.

### 3.3 Impact of other eicosanoid pathways

Different eicosanoid receptor pathways can have distinct, and often opposing, biological functions. For example, LTB_4_-BLT1/2 and cysteinyl leukotriene-CysLT1/2 pathways induce neutrophilic and asthmatic inflammatory responses, respectively.^97-100^ In a colorectal cancer model (Apc^Min/+^ mice), loss of LTB_4_-BLT1 signaling was associated with increased Verrucomicrobia (*Akkermansia muciniphila*) and Firmicutes and decreased Bacteroidetes.^101^ Interestingly, BLT1-deficiency-driven microbiota changes were MyD88-independent, and BLT1 and MyD88 might act synergistically to control *Akkermansia.*^101^

Other eicosanoids also influence barrier function and permeability. PGD_2_ and its metabolite 15d-PGJ_2_ decrease intestinal permeability.^102-104^ While LTB_4_-BLT1 signaling maintains intestinal barrier integrity by preserving tight junction proteins,^101^ other 5-LOX metabolites, such as LTC_4_, LTD_4_ and 5-HETE, can increase intestinal paracellular permeability by disrupting tight junctions, potentially via PLC/PKC activation.^105^^,^^106^ Finally, although many eicosanoids are unlikely to directly bind nuclear receptors, some (like PGE_2_) can modulate nuclear receptor transcriptional activities in various cell types,^107-109^ providing an additional layer of context-dependent regulation.

Overall, eicosanoid-microbiota interactions are highly context dependent, reflecting differences in ligand availability, receptor expression, and the local tissue environment. Challenges remain to identify the dominant eicosanoid pathways that control microbiota in specific physiological or disease settings. Another warrant is to determine to what extent microbiota shifts are driven by host immunity and barrier programs. Establishing these mechanisms is essential to explaining how disease-specific outcomes occur.

## 4 Microbiota-eicosanoid crosstalk in chronic inflammatory diseases

### 4.1 IBD

**Figure 3.**
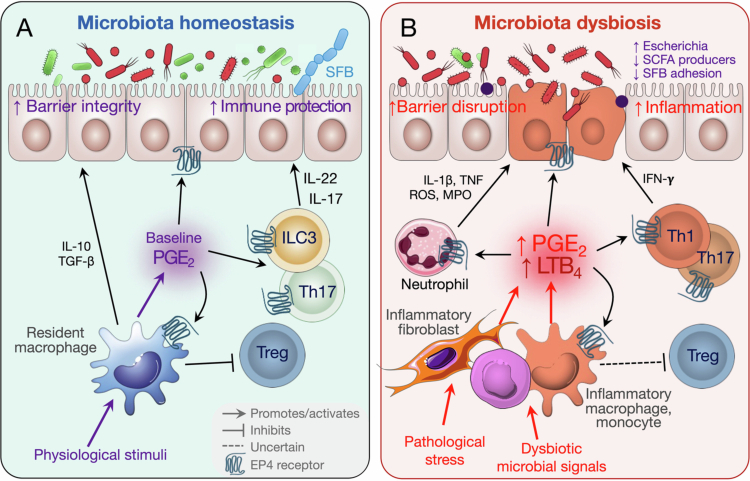
Microbiota–eicosanoid interactions in IBD. (A) Under physiological conditions, basal PGE_2_ supports epithelial integrity and confers immune protective effects through EP4-mediated actions on epithelial cells, ILC3s (and possibly Th17 cells), and gut-resident macrophages. This helps maintain a stable commensal community. PGE_2_ also restrains intestinal Treg responses, contributing to the steady-state balance of mucosal immunity. (B) Pathogenic stress and dysbiotic microbial cues drive increased production of PGE_2_ and other inflammatory eicosanoids such as LTB_4_ by fibroblasts and myeloid cells. Elevated eicosanoid signaling promotes epithelial injury and fuels mucosal inflammation by enhancing pathogenic Th1 and/or Th17 responses, activating inflammatory macrophages and monocytes, and recruiting neutrophils. These effects may be further amplified via suppression of Tregs. These host inflammatory responses reinforce dysbiosis, including expansion of *Escherichia* species, loss of beneficial SCFA-producing taxa, and reduced SFB adhesion, leading to sustained barrier breakdown and chronic inflammation.

IBD is a chronic immune-mediated inflammatory disorder of the gastrointestinal tract, primarily including CD and UC. Its pathogenesis arises from a complex interplay between genetic susceptibility, dysregulated immune functions, alterations in the gut microbiota, and environmental triggers. In IBD, microbiota-driven innate sensing together with dysregulated PUFA metabolism skews COX/LOX-dependent eicosanoid production. In turn, these lipid mediators reshape microbiota communities by affecting epithelial barrier function and mucosal immune tone, establishing positive feedback loops that fuel ongoing inflammation. Consistent with this, microbial dysbiosis is linked to disturbed intestinal eicosanoid metabolism, especially during inflammation. Conversely, eicosanoid-driven changes in the mucosal environment can further remodel the microbiota and influence disease course. While immune dysregulation and epithelial barrier dysfunction have long been recognized as central features of IBD, emerging evidence suggests that profound metabolic reprogramming, particularly within microbiota-eicosanoid crosstalk, plays a key role in sustaining and amplifying intestinal inflammation (Figure 3).

#### 4.1.1 The COX pathway

Beyond serving as substrates for eicosanoid biosynthesis, dietary fatty acids can also modulate intestinal inflammation through receptor-mediated pathways. For example, PUFAs associated with Western-style diets can promote Crohn’s-like inflammation by inducing epithelial RXR activities.^110^ In addition, the *ω*-3/ω-6 PUFA balance helps maintain intestinal barrier function and mucosal homeostasis, and disruption of this balance has been linked to IBD and metabolic disease.^111^^,^^112^ In both murine models and human intestinal inflammation, the expression of key eicosanoid-synthesizing enzymes, such as COX-1/2 and mPGES, is markedly upregulated, resulting in increased production of PGE_2_ and other lipids capable of activating the EP4 receptor. For example, in dextran sodium sulfate- and 2,4,6-trinitrobenzenesulfonic acid-induced experimental models for UC, PGE synthase gene expression and associated PGE_2_ production are significantly increased in intestinal and mesenteric lymphatic tissues.^113^ Similar upregulation of PGE_2_ synthases, elevated PGE_2_ levels, and altered production of other eicosanoids have also been reported in T cell-driven animal models for CD.^16^ Consistently, increased upregulation of core PGE synthase genes and PGE_2_ production were also seen in human UC and CD intestine and blood samples.^16^^,^^17^^,^^114^ In the intestine, stromal fibroblasts and myeloid cells, especially monocytes and macrophages, are major producers of eicosanoids such as PGE_2_.^16^^,^^37^ In stromal cells, eicosanoid synthesis is typically driven by tissue-derived cues including cytokines,^115^^,^^116^ whereas in myeloid cells it is strongly influenced by microbial stimuli from the intestinal lumen, encompassing both pathogens and commensals.^35^ By contrast, epithelial cells and other non-myeloid populations generally contribute relatively little to the eicosanoid pool at steady state. However, under epithelial stress or inflammatory signaling, epithelial eicosanoid production can increase and act locally to influence epithelial repair and barrier function.

Under homeostatic conditions, the microbiota-COX-PGE_2_-EP4 axis supports mucosal integrity and regulates immune responses (Figure 3A). This pathway can be initiated by specific commensal or probiotic strains, such as *Lactobacillus rhamnosus GG*, which stimulate MyD88-dependent, COX-2-driven PGE_2_ production to promote epithelial restitution.^37^ Acting on intestinal epithelial cells, PGE_2_-EP4 signaling enhances survival and regeneration via the cAMP-PI3K-ERK pathways, thereby reducing apoptosis, promoting proliferation, and preserving goblet cell populations to restore barrier integrity.^117^ EP4 activation in epithelial cells also suppresses TNF-induced necroptosis by inhibiting the RIPK1–MLKL cascade^118^ and accelerates wound repair by inducing wound-associated epithelial cell differentiation through a non-canonical Wnt pathway involving GSK-3β inhibition and nuclear *β*-catenin accumulation.^119^

Beyond the epithelium, the PGE_2_-EP4 pathway coordinates repair and immune regulation across stromal and immune compartments. In the stromal-lymphatic niche, EP4 promotes lymphangiogenesis via the VEGF-VEGFR3 signaling,^120^ and gut microbial stimulation of lymphatic endothelial cells can recruit immature myeloid cells that secrete COX-2-derived PGE_2_. This eicosanoid acts through EP4 to further enhance lymphangiogenesis and induce regenerative factors such as R-spondin 3, supporting intestinal epithelial repair and regeneration.^121^ In parallel, bacterial or inflammatory cues activate a TPL2-ERK-COX-2-PGE_2_-EP4 cascade in intestinal myofibroblasts, reinforcing epithelial proliferation.^122^ Immunologically, EP4 signaling in ILC3s boosts the production of IL-22 and antimicrobial peptides (e.g., Reg3β and Reg3γ).^77^ In T cells, PGE_2_ promotes type 17 T helper (Th17) cell differentiation and expansion, and Th17-driven IL-17 plays protective roles similar to IL-22 in maintaining intestinal epithelial homeostasis.^123^ PGE_2_-EP4 signaling has also been implicated in shaping adaptive immunity, with enteroprotective effects linked to regulation of Treg function and suppression of Th1 cell activation.^124^^,^^125^

In addition to effects in epithelial, stromal, and lymphoid cells, the PGE_2_-EP4 axis is likely to be particularly important in myeloid cells, given that they are among the dominant intestinal sources of PGE_2_ and express high levels of EP4, positioning them to both generate and respond to this signal within the gut. Selective deletion of mPGES1, the terminal enzyme for PGE_2_ biosynthesis, in non-lymphoid cells reduced Treg abundances in both mesenteric lymph nodes and the colon, expanded inflammatory CD4^+^ T cells, and worsened T cell-driven colonic inflammation.^124^ Mechanistically, PGE_2_-EP4 signaling in macrophages/monocytes can limit gut injury and promote tissue repair, in part through regulating IL-10 and TGF-*β* signaling. For example, Na et al. showed that EP4 activation in CSF1R^+^ macrophages promoted CXCL1 production, which in turn drives epithelial differentiation and proliferation in regenerating crypts during dextran sulfate sodium (DSS)-induced colitis, thereby enhancing mucosal repair.^75^ Consistent with a protective EP4 axis in macrophages, Nakatsuji et al. reported that macrophage-specific deficiency of EP4-associated protein (EPRAP), a cytoplasmic EP4-interacting molecule required for EP4-driven downstream cAMP signaling, increased multiple pro-inflammatory mediators (e.g., CXCL1, MCP-1, IL-6, and IL-1β) and worsened DSS-induced colitis and inflammation-associated colorectal cancer, whereas macrophage-specific EPRAP overexpression ameliorated DSS colitis.^126^ In line with these findings, we and others have further observed that EP4 deletion in additional Cre-driven macrophage lineages (e.g., CD11c-Cre and CX_3_CR1-cre) similarly exacerbated DSS-induced colitis (reference^127^ and unpublished observations). These findings from animal models are consistent with recent genetic and single-cell network analyses showing that a *PTGER4* (encoding EP4)-containing co-expression meta-module of UC risk genes in healthy macrophages may be linked to cAMP-related signaling pathways that could influence macrophage activation.^127^^,^^128^

On the other hand, dysregulated or sustained COX-PGE_2_-EP4 signaling can exacerbate intestinal inflammation by engaging immunological epithelial and microbial pathways (Figure 3B). PGE_2_ can reinforce microbiota dysbiosis through EP4-dependent suppression of type I interferon production in mononuclear phagocytes and limitation of intestinal Treg expansion and accumulation, establishing a pathogenic feed-forward loop.^17^ In parallel, EP4 can also directly act on CD4^+^ T cells to bias differentiation toward inflammatory effector lineages. For example, PGE_2_, signaling through EP2 and EP4 receptors, directly inhibits Treg differentiation and functions. Mice with a specific knockout of EP2 or EP4 in CD4^+^ T cells or Foxp3^+^ Tregs show higher Treg frequencies in the intestine and peripheral lymph nodes, and they experience less intestinal inflammation in different colitis models.^129-131^ Within the tumor microenvironment, however, PGE_2_-EP2/EP4 signaling has also been observed to encourage Treg induction and maintain Treg phenotype.^132^^,^^133^ PGE_2_-EP4 signaling has been shown to promote Th1 and Th17 polarization and effector function, increase their accumulation in the intestine, and amplify mucosal inflammatory responses.^124^^,^^129^^,^^130^^,^^134^ Consistent with a clinically relevant role for this pathway, host–microbe co-evolution analyses of IBD genetic risk loci identified *PTGER4* among genes whose IBD-associated variation bears signatures of selective pressure linked to microbial exposure, supporting a role for altered EP4 signaling in shaping mucosal immune-microbial homeostasis.^135^

At the barrier level, although PGE_2_ is well-known to critically maintain intestinal integrity, but it can also impair intestinal integrity under certain conditions. Clinically, intestinal COX-2–PGE_2_–EP4 pathway gene expression was elevated at baseline in IBD patients compared with non-IBD controls and was highest in IBD patients resistant to anti-TNF therapy relative to both anti-TNF responders and non-IBD patients. Following anti-TNF treatment, COX-2–PGE_2_–EP4 signaling decreased in responders, whereas it failed to decline and remained persistently high in non-responders.^16^ These findings link enhanced and sustained activation of the COX-2–PGE_2_ pathway in anti-TNF failure with heightened intestinal inflammation and impaired epithelial regeneration, potentially through suppression of stem cell function, providing a plausible mechanism for treatment resistance in ulcerative colitis.^136^ Loss of IL-10 signaling in macrophages increased expression of PGE_2_-EP4 pathway genes, elevated PGE_2_ production, and impaired macrophage bacterial killing.^137^ Collectively, these findings suggest that context-dependent modulation of PGE_2_-EP4 signaling may help rebalance microbiota–eicosanoid interactions and control amplified inflammation in IBD. Therapeutically, targeting the COX- PGE_2_-EP4 axis can mitigate intestinal inflammation in experimental settings. COX inhibitors reduce colitis severity in animal models, at least in part by limiting PGE_2_ overproduction.^138^ In addition, microbiota-derived factors (e.g., HM0539 from *L. rhamnosus GG*) and microbiota-modulating agents such as berberine have been reported to alleviate colitis by suppressing the COX-2–PGE_2_ signaling.^139^^,^^140^

#### 4.1.2 The LOX pathway

Enhanced 5-LOX activity and increased LTB_4_ production represent a major proinflammatory pathway that links microbial sensing to immune activation in IBD. Elevated 5-LOX activity and LTB_4_ levels have been reported in inflamed colonic tissue and in leukocytes from IBD patients.^141-143^ Microbial PRR signaling, particularly Dectin-1 activation in intestinal macrophages, upregulates 5-LOX and LTA_4_ hydrolase and promotes LTB_4_ biosynthesis and inflammasome-dependent IL-1β production, thereby exacerbating tissue injury.^144^ In contrast, disruption of microbial recognition further perturbs bacterial community structure and ameliorates colitis severity.^145^ LTB_4_ can also amplify inflammation by reinforcing microbial dysbiosis. For example, BLT1 deficiency reshapes gut microbiota composition, including enrichment of *Akkermansia muciniphila*, but impairs antimicrobial responses, leading to MyD88-dependent pro-inflammatory cytokine production, COX-2 induction, and enhanced susceptibility to colitis and tumorigenesis.^101^ As a key neutrophil chemoattractant, the increased neutrophil chemotactic activity observed in IBD mucosa is largely attributable to LTB_4_.^146^ Beyond recruitment, LTB_4_ also acts as a neutrophil-derived secondary amplifier that enhances the magnitude of neutrophil transepithelial migration initiated by epithelial hepoxilin A3.^147^ Consistently, pharmacological blockade of LTB_4_ signaling reduces neutrophil infiltration and oedema in experimental colitis, supporting a pathogenic role for this pathway.^148^ Similarly, intestinal manipulation was shown to induce 5-LOX-dependent LTB_4_ production and leukocyte infiltration in the muscularis externa, contributing to postoperative ileus, and these effects were ameliorated by 5-LOX-deficiency or antagonism.^149^ Collectively, the 5-LOX-LTB_4_-BLT1 axis emerges as a key microbiota-responsive amplifier of IBD pathology, representing a potential therapeutic target to control intestinal inflammatory responses.

### 4.2 Metabolic diseases

**Figure 4.**
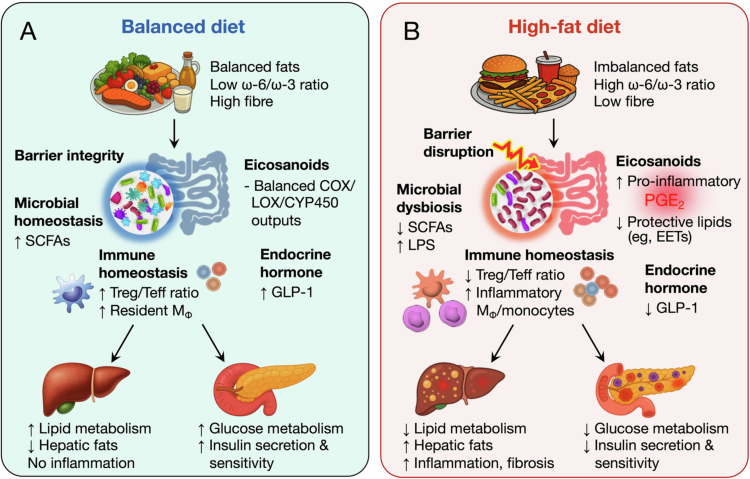
Microbiota–eicosanoid interactions in metabolic diseases. (A) A diet with balanced fatty acids (low *ω*-6/ω-3 PUFA ratio) and high fiber helps maintain gut epithelial barrier integrity, microbial homeostasis, a balanced eicosanoid profile, normal endocrine hormone levels (e.g., GLP-1), and immune equilibrium. These coordinated effects support metabolic health, including efficient lipid and glucose metabolism and intact insulin signaling. (B) A high-fat diet, particularly when combined with an imbalanced fat composition such as an elevated *ω*-6/ω-3 PUFA ratio, disrupts gut barrier function, induces microbial dysbiosis, disturbs immune and eicosanoid balance, and reduces protective endocrine hormones. These changes impair lipid and glucose metabolism, promote hepatic inflammation, and contribute to the development of metabolic diseases, including NAFLD/NASH and insulin-resistant diabetes.

Metabolic diseases are characterized by impaired glucose and lipid homeostasis, chronic low-grade systemic inflammation, and insulin resistance. Diabetes mellitus features persistent hyperglycemia due to impaired insulin secretion and/or reduced insulin sensitivity, leading to widespread disturbances in whole-body energy metabolism. Non-alcoholic fatty liver disease (NAFLD), the hepatic manifestation of metabolic syndrome, ranges from simple steatosis to non-alcoholic steatohepatitis (NASH), which is associated with hepatocellular damage, inflammation, and fibrosis. These interlinked conditions share key features, including abnormal lipid accumulation and reduced metabolic flexibility, underscoring an important role for gut microbiota–lipid interactions in metabolic dysfunction. In metabolic disease, gut dysbiosis and barrier dysfunction promote metabolic endotoxemia, linking microbial signals to systemic inflammation and insulin resistance. In parallel, metabolic stress and microbial cues reprogramme hepatic eicosanoid pathways, particularly the CYP450-EET-sEH axis, affecting steatohepatitis progression, fibrosis, and glucose homeostasis. Dietary fats shape these processes by supplying substrates for eicosanoid biosynthesis and modulating host-microbial energy metabolism, altering the intestinal milieu and downstream metabolic outcomes (Figure 4).

#### 4.2.1 Gut microbiota dysbiosis and metabolic endotoxemia

One hallmark of metabolic dysfunction is gut microbial dysbiosis, which depletes SCFA producers and enriches pro-inflammatory LPS-linked taxa, promoting endotoxemia, inflammation, and insulin resistance. Patients with type 2 diabetes (T2D) mellitus commonly exhibit a decrease in butyrate-producing taxa (e.g., *Faecalibacterium* and *Roseburia*) alongside an expansion of opportunistic LPS-producing bacteria. These compositional changes are accompanied by reduced SCFA levels and increased systemic LPS levels, which together promote chronic, low-grade inflammation and insulin resistance.^150-153^ Similarly, patients with NAFLD/NASH show an increased abundance of pro-inflammatory taxa (e.g., *Bacteroides*, *Ruminococcus*, *Streptococcaceae*, *Enterobacteriaceae*, and *Veillonella*) and reduced abundance of potentially beneficial taxa (e.g., *Prevotella*, *Lactobacillaceae*, and *Bifidobacterium*).^154^ Importantly, microbial shifts in NAFLD/NASH correlate with histological severity, and the enrichment of certain genera (e.g., *Bacteroides*, *Ruminococcus*) has been associated with NASH and more advanced fibrosis.^155^ Microbiota-derived SCFAs shape host metabolism by modulating enteroendocrine hormone secretion and attenuating inflammatory responses, thereby influencing both glucose and lipid homeostasis.^156^^,^^157^ Accordingly, dysbiosis-associated SCFA depletion has been linked to impaired glucose regulation and augmented susceptibility to metabolic disorders.^158^ In rodent models, interventions that restore SCFA-producing bacteria and subsequently SCFA levels can improve metabolic outcomes.^159^

Metabolic endotoxemia is characterized by elevated circulating LPS, largely arising from increased intestinal permeability, and is strongly associated with high-fat diet consumption and microbial dysbiosis.^158^^,^^160^ Following intestinal bacterial overgrowth and/or translocation, increased circulating LPS activates the TLR4/CD14 axis, promoting systemic, low-grade chronic inflammation and insulin resistance in T2D mellitus.^161-164^ Consistently, fasting LPS levels are significantly increased in patients with T2D mellitus,^165^ and endotoxemia predicts an increased risk of developing diabetes.^166^ In NAFLD, endotoxemia similarly contributes to hepatic inflammation,^167^^,^^168^ as reflected by elevated plasma endotoxin levels, which correlate positively with hepatic steatosis severity and disease progression.^169^^,^^170^ Moreover, low-dose LPS also sustains low-grade activation of p38 MAPKs and neutrophil infiltration, exacerbating high-fat diet-induced steatohepatitis.^171^ Therapeutic modulation of the gut microbiota (e.g., with probiotics or antibiotics) can reduce endotoxemia, rebalance eicosanoid profiles, improve gut barrier function, and attenuate metabolic inflammation in both clinical and experimental settings.^20^^,^^172^^,^^173^

#### 4.2.2 Dysregulation of the CYP450-EET-sEH pathway

Hepatic eicosanoid metabolism is profoundly dysregulated in metabolic disease, particularly with alterations in the CYP450–EET–sEH axis. EETs exert beneficial effects on glucose homeostasis by supporting pancreatic islet cell function and enhancing peripheral insulin sensitivity.^174^ In addition to host enzymes, the gut microbiota contributes to colonic lipid metabolism by converting EETs into their corresponding diols.^20^ Specifically, commensal bacteria-derived dihydroxy fatty acids, the downstream sEH pathway products (e.g., 9,10-DiHOME), facilitate the differentiation of regulatory T cells.^175^ In type 1 diabetes mellitus, EETs protect pancreatic *β*-cells from cytokine-induced apoptosis by inhibiting NF-κB activation and nitric oxide production.^176^ Increasing EET biosynthesis, for example via CYP2J3 gene therapy, improves insulin sensitivity and reduces blood pressure in diabetic rodents, partly by enhancing insulin receptor signaling and activating AMPK in peripheral tissues.^177^ Conversely, sEH promotes insulin resistance and reduces islet size in high-fat diet-induced T2D mellitus.^174^ Furthermore, sEH inhibition has been shown to alleviate high-sucrose diet-induced colonic inflammation and bolster tight junction integrity, highlighting it as a potential therapeutic approach for gut barrier dysfunction.^178^

Beyond diabetes, this pathway is also pivotal in NASH and NAFLD pathogenesis. In pediatric NAFLD, hepatic EET levels increase during steatosis (consistent with reduced sEH activity) but decline as fibrosis develops (reflecting diminished CYP450 epoxygenase expression), suggesting a stage-dependent protective role.^179^ While high-fat diet-upregulated sEH facilitates disease progression,^180^ sEH inhibition attenuates hepatic inflammation and steatosis.^181^ Specifically, sEH inhibition attenuates chronic ethanol-induced liver injury by increasing EpFA levels and reshaping the gut microbiota, but it fails to enrich *Akkermansia* in ethanol-fed mice.^182^ Moreover, sEH deficiency or pharmacological inhibition alleviates diet-induced endoplasmic reticulum stress in liver and adipose tissue, improves insulin signaling, and attenuates carbon tetrachloride-induced hepatic fibrosis, highlighting sEH as a promising therapeutic target in metabolic syndrome and liver fibrosis.^183^^,^^184^

#### 4.2.3 Therapeutic microbiota–eicosanoid interventions

Fecal microbiota transplantation (FMT) can restore microbial homeostasis and shows therapeutic promise in metabolic diseases. In NAFLD/NASH, FMT reduces hepatic fat accumulation and attenuates steatohepatitis by correcting gut microbiota dysbiosis, with clinical efficacy appearing more pronounced in lean than in obese phenotypes.^185-188^ In T2D mellitus, FMT improves glycemic control, lipid profiles, and insulin resistance by reshaping the gut microbiota, alleviating the hyperglycemia, and reversing insulin resistance and reducing body mass index through enriching beneficial taxa, including *Chlorobium phaeovibrioides*, *Bifidibacterium adolescentis*, and *Synechococcus sp.WH8103.*^189-191^ Repeated FMT in obese patients with T2D mellitus has been shown to promote sustained engraftment of lean donor microbiota, and combining FMT with lifestyle interventions further enriches beneficial probiotics and improves lipid profile and liver stiffness.^192^ Moreover, FMT may ameliorate T2D mellitus by remodeling the gut communities in ways that shift their serum metabolites, reinforce gut barrier function, and suppress systemic inflammatory immune responses.^193^

These effects at least partially reflect FMT-driven changes in microbial metabolites and lipid signaling. Microbial metabolites such as SCFAs enhance insulin secretion and sensitivity, strengthen the intestinal barrier, and suppress chronic inflammation through their receptors including GPR41, GPR43 and GPR109A.^194-197^ Accordingly, dietary SCFA supplementation and pharmacological strategies that increase SCFA availability can ameliorate insulin resistance, hepatic steatosis, and NASH progression through activating AMPK and GLP-1 signaling, and also enhance gut barrier function via the anti-inflammatory pathway.^197-200^ Dietary PUFAs similarly intersect with the microbiota-eicosanoid axis. Supplementation with *ω*-3 PUFA increases pro-resolving lipid mediators,^201^ modulates gut microbiota composition,^202^^,^^203^ and alleviates NAFLD/NASH progression.^204^ In addition, gut microbiota-dependent PUFA metabolism (e.g., conversion of dietary linoleic acid to bioactive metabolites such as HYA) improves glucose homeostasis and promotes intestinal peristalsis, thereby reducing lipid absorption and adipose inflammation.^25^ Consistently, high-fiber dietary intervention reduces liver steatosis, improves PUFAs profiles, and lowers PGE_2_ levels in patients with NAFLD.^205^

Chronic low-grade inflammation can drive COX-2 upregulation and sustained PGE_2_ production, aggravating metabolic dysfunction. Clinically, circulating PGE_2_ metabolites correlate with T2D milieu status and therapeutic response.^206^ Pharmacological COX-2 inhibition ameliorates hepatic inflammation and improves insulin resistance in NASH by suppressing the non-canonical Wnt5a/JNK1 pathway.^207^ However, genetic deletion of mPGES-1 worsens NASH-associated liver inflammation and hepatocyte apoptosis by disrupting PGE_2_-mediated suppression of macrophage TNF-*α* production, suggesting that blanket inhibition of PGE_2_ biosynthesis may be context-dependent and that other PGs may also shape disease biology.^208^ Accordingly, targeting downstream PG receptors (e.g., IP or EP3) is likely to provide a more selective means to regulate liver inflammation, fibrosis, and pancreatic *β*-cell dysfunction.^209^^,^^210^

### 4.3 Arthritis–RA, SpA

**Figure 5.**
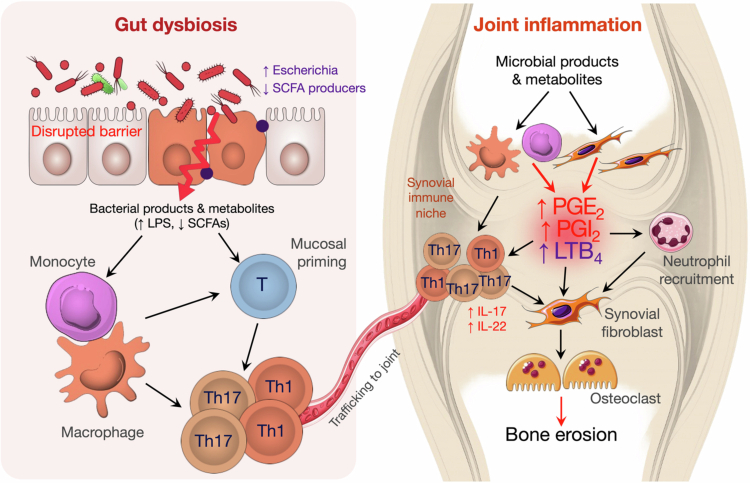
Microbiota-eicosanoid interactions along the gut-joint axis in arthritis. Gut microbial dysbiosis activates inflammatory myeloid cells through altered bacterial products and metabolites, such as increased LPS and reduced SCFAs, driving the differentiation of pathogenic Th1 and Th17 cells and promoting their migration to the joint. Within the synovial niche, circulating microbial products stimulate local fibroblasts and myeloid cells to generate high levels of pro-inflammatory eicosanoids (including PGE_2_, PGI_2_, and LTB_4_). These mediators amplify joint inflammation by expanding pathogenic T cells, activating inflammatory macrophages/monocytes, and recruiting neutrophils. Altogether, these signals promote synovial fibroblast activation and osteoclast differentiation, leading to bone erosion and chronic joint inflammation.

Arthritis, notably rheumatoid arthritis (RA) and spondyloarthritis (SpA), is an immune-mediated joint disorder driven by distinct yet partially overlapping immunopathological pathways. RA typically presents with symmetric polyarthritis and synovial hyperplasia, maintained by pro-inflammatory cytokine networks (e.g., TNF-*α* and IL-6) and autoantibody-associated immune responses. By contrast, SpA is marked by predominant axial and entheseal inflammation and is often accompanied by extra-articular manifestations. Despite these clinical differences, both conditions reflect a complex interplay of genetic susceptibility, environmental triggers, and mucosal immune dysregulation. In inflammatory arthritis, gut microbiota dysbiosis and alterations in microbial metabolites can drive mucosal immunity and promote systemic immune activation, contributing to joint inflammation. Within the joint, COX- and LOX-derived eicosanoid pathways help set the inflammatory tone, linking gut-driven immune perturbations to synovial inflammation, neutrophil recruitment, and bone remodeling (Figure 5). These observations together suggest that microbiota-eicosanoid crosstalk links intestinal mucosal dysregulation to the immune processes that drive joint pathology.

#### 4.3.1 Gut microbial dysbiosis in arthritis

Beyond intestinal inflammation and metabolic inflammation (including liver diseases and diabetes mellitus), chronic inflammation in distal tissues, such as the joints, can also reciprocally influence the gut microbiota and promote dysbiosis. In RA and SpA, disease-associated gut microbial dysbiosis reshapes microbial taxa and metabolite profiles, thereby engaging mucosal and joint immune pathways that modulate inflammation.

In RA, expansion of *Prevotella copri* frequently coincides with depletion of *Bacteroides* and other beneficial taxa. Additional RA-associated microbes include *Fusobacterium nucleatum, Eggerthella lenta, Escherichia coli, Collinsella, Lactobacillus,* and *Streptococcus.*^211-215^ Mechanistic evidence further supports a pathogenic role for specific taxa. For example, *Fusobacterium nucleatum* can aggravate disease via FadA-containing outer membrane vesicles,^216^ and *Subdoligranulum* is targeted by autoantibodies in at-risk individuals.^217^ Integrated microbiome-metabolome analyses have also revealed enrichment of *Klebsiella* and *Escherichia* and depletion of *Fusicatenibacter,* accompanied by disrupted tryptophan and glycerophospholipid metabolism.^218^

In SpA, disease activity correlates with the abundance of *Ruminococcus gnavus*, *Clostridium bolteae, Clostridium symbiosum*, and *Dialister.*^215^^,^^219^^,^^220^ Among HLA-B27^+^ ankylosing spondylitis patients, increased *Faecalibacterium prausnitzii* and *Coprococcus* and decreased *Bacteroides fragilis*, *Ruminococcus*, and *Akkermansia muciniphila have been reported.*^221^ Similarly, reductions in *Ruminococcus* and *Akkermansia* distinguish psoriatic arthritis (PsA) from healthy controls and from psoriasis patients without arthritis.^222^

Across RA and SpA, a common functional signature is the loss of SCFA-producing bacteria and reduced butyrate availability. Butyrate-producing taxa such as *Bifidobacterium* in RA and *Faecalibacterium prausnitzii* in SpA are markedly decreased.^212^^,^^223^^,^^224^ RA is additionally characterized by enrichment of butyrate-consuming species, which also contributes to lower butyrate levels and facilitates joint autoimmunity and bone erosion.^225^ In line with this, higher SCFAs levels correlate with non-progression to arthritis in at-risk of individuals, whereas declining butyrate appears to precede the clinical onset of RA and PsA.^226^^,^^227^

These microbial and metabolic factors are linked to mucosal immune dysregulation, particularly Th17 polarization and dissemination, which connects intestinal inflammation to joint pathology. Expansion of pro-inflammatory Th17 cells strongly correlates with systemic disease activity in RA.^228^ *Subdoligranulum didolesgii* translocate across the intestinal epithelium and directly trigger Th17 cell responses,^217^ while SFB potently induce small intestinal Th17 cells and facilitate their systemic dissemination.^229-231^ Additionally, IgA-coated *E. coli* in CD-associated SpA promotes Th17-mediated inflammation, further supporting a gut-joint immune axis.^232^

Finally, functional studies and interventions support a causal contribution of SCFAs to disease modulation. SCFAs attenuate disease severity in HLA-B27 and β2-microglobulin transgenic models and in experimental arthritis,^233^^,^^234^ increase bone volume, and suppress osteoclastogenesis in K/BxN and collagen-induced arthritis models.^235^ Consistent with these mechanisms, high-fiber dietary interventions increase systemic SCFAs, reduce pro-inflammatory mediators, and improve the functional outcomes in clinical settings.^236^^,^^237^

#### 4.3.2 The COX Pathway

Microbial dysbiosis and associated metabolic shifts can also influence eicosanoid pathways within the joint microenvironment. In RA, COX-2 is upregulated, and prostaglandin production, especially PGE_2_ and PGI_2_, is increased. These mediators stimulate IL-6 production from synovial fibroblasts and promote Th1/Th17 activation via the EP4, EP2, and IP receptors, thereby amplifying joint inflammation.^238^^,^^239^ In SpA, PGE_2_-EP4 signaling is linked to radiographic progression by enhancing pathogenic bone formation via interactions between CD14^high^EP4⁺ cells and mesenchymal stem cells.^240^ Notably, EP4 signaling exerts context-dependent effects on bone remodeling, but in inflammatory settings, it can promote osteoclastogenesis and osteolysis by inducing RANKL (receptor activator of NF-κB ligand) expression in stromal/fibroblastic and osteoblastic cells, activating the RANKL-RANK axis.^241-243^ In parallel, PGE_2_ signaling via EP4/EP2-cAMP pathway can further drive bone resorption matrix degradation, in part through induction of matrix metalloproteinases.^244^^,^^245^ Clinically, targeting this pathway remains highly relevant. COX-2 inhibition is a first-line treatment strategy for inflammatory joint symptoms, with celecoxib providing effective symptom control in RA and ankylosing spondylitis,^246^^,^^247^ while EP4 antagonism suppresses inflammatory T-cell responses, alleviates pain, reduces disease severity,^248^ and attenuates RANKL-induced osteoclast differentiation and bone resorption.^249^ Collectively, dysbiosis-linked activation of the COX-2-PGE_2_-EP4 axis provides a mechanistic bridge between altered microbial metabolism, joint inflammation, and bone remodeling, but it remains a tractable therapeutic target in inflammatory arthritis.

#### 4.3.3 The LOX Pathway

LTB_4_ biosynthesis is governed by the coordinated activity of 5-LOX and 5-LOX-activating protein. 5-LOX is predominantly expressed in RA synovial macrophages, neutrophils, and mast cells.^250^ Across inflammatory arthritis, LTB_4_ signaling has been implicated in both RA and PsA. Elevated LTB_4_ production and oxidative stress contribute to PsA progression^251^ and may serve as a predictive biomarker for PsA development.^226^ Mechanistically, LTB_4_ amplifies synovial inflammation by inducing inflammatory cytokines (e.g., TNF-*α*, IL-1β, IL-32, IFN-*γ*) and chemokines in synovial fibroblasts and immune cells, while also promoting synovial cell apoptosis.^252^^,^^253^ In addition, IL-23-driven LTB_4_ activates BLT1/BLT2 receptors on osteoclast precursors, triggering PLC-dependent calcium signaling and NFAT activation, thereby inducing osteoclastogenic gene expression and driving bone erosion.^254^ Consistent with these mechanisms, pharmacological inhibition of 5-LOX or 5-LOX-activating protein suppresses pro-inflammatory leukotriene production and exhibits anti-inflammatory efficacy, and targeting this pathway limits joint destruction in a murine model.^255^^,^^256^ However, despite correlations between neutrophil LTB_4_ release and disease activity, an LTB_4_ antagonist, BIIL 284, showed limited efficacy in human RA trials, highlighting a complex and compensatory inflammatory landscape.^257^^,^^258^ Overall, the 5-LOX-LTB_4_ pathway provides a strong mechanistic link between synovial inflammation and bone erosion, but the clinical benefit of targeting this pathway requires further evaluation.

## 5 Conclusion

The bidirectional interplay between gut microbiota and eicosanoid signaling plays a critical role in the pathogenesis of chronic inflammatory diseases such as IBD, metabolic disorders, and arthritis. The dysregulation of this axis perpetuates inflammation and tissue damage. Current challenges include the context-dependent functions of key mediators like the EP4 receptor, the complexity of host-microbe interactions, and the risk of side effects from targeting broadly active pathways such as COX and LOX. A deeper understanding is needed of how microbiota dysbiosis, microbial products and metabolites, host bioactive lipid signaling pathways, and the host immune system are integrated across inter-kingdom and inter-organ networks in both homeostatic and chronic inflammatory conditions. It is also important to clarify how these interactions are shaped by external inputs, such as diets and medications. Future research should focus on elucidating cell-type and context-specific mechanisms, developing targeted therapies that selectively modulate pro-resolving pathways without disrupting homeostasis, and exploring personalized interventions guided by individual microbiota and lipid mediator profiles. Integrating multi-omics data with clinical outcomes will be essential to translate these insights into effective, safe, and precision-based anti-inflammatory strategies.

## Data Availability

Not applicable.

## References

[1] Gilbert JA, Blaser MJ, Caporaso JG, Jansson JK, Lynch SV, Knight R. Current understanding of the human microbiome. Nat Med. 2018;24:392–400. doi: 10.1038/nm.4517. 29634682 PMC7043356

[2] Fan Y, Pedersen O. Gut microbiota in human metabolic health and disease. Nat Rev Microbiol. 2021;19:55–71. doi: 10.1038/s41579-020-0433-9. 32887946

[3] de Vos WM, Tilg H, Van Hul M, Cani PD. Gut microbiome and health: mechanistic insights. Gut. 2022;71:1020–1032. doi: 10.1136/gutjnl-2021-326789. 35105664 PMC8995832

[4] Iliev ID, Ananthakrishnan AN, Guo CJ. Microbiota in inflammatory bowel disease: mechanisms of disease and therapeutic opportunities. Nat Rev Microbiol. 2025;23:509–524. doi: 10.1038/s41579-025-01163-0. 40065181 PMC12289240

[5] Lynch SV, Pedersen O. The human intestinal microbiome in health and disease. N Engl J Med. 2016;375:2369–2379. doi: 10.1056/NEJMra1600266. 27974040

[6] Di Vincenzo F, Del Gaudio A, Petito V, Lopetuso LR, Scaldaferri F. Gut microbiota, intestinal permeability, and systemic inflammation: a narrative review. Intern Emerg Med. 2024;19:275–293. doi: 10.1007/s11739-023-03374-w. 37505311 PMC10954893

[7] Chae YR, Lee YR, Kim YS, Park HY. Diet-induced gut dysbiosis and leaky gut syndrome. J Microbiol Biotechnol. 2024;34:747–756. doi: 10.4014/jmb.2312.12031. 38321650 PMC11091682

[8] Yang W, Cong Y. Gut microbiota-derived metabolites in the regulation of host immune responses and immune-related inflammatory diseases. Cell Mol Immunol. 2021;18:866–877. doi: 10.1038/s41423-021-00661-4. 33707689 PMC8115644

[9] Medzhitov R. Origin and physiological roles of inflammation. Nature. 2008;454:428–435. doi: 10.1038/nature07201. 18650913

[10] Hotamisligil GS. Inflammation, metaflammation and immunometabolic disorders. Nature. 2017;542:177–185. doi: 10.1038/nature21363. 28179656

[11] Furman D, Campisi J, Verdin E, Carrera-Bastos P, Targ S, Franceschi C, Ferrucci L, Gilroy DW, Fasano A, Miller GW, et al. Chronic inflammation in the etiology of disease across the life span. Nat Med. 2019;25:1822–1832. doi: 10.1038/s41591-019-0675-0. 31806905 PMC7147972

[12] Yao C, Narumiya S. Prostaglandin-cytokine crosstalk in chronic inflammation. Br J Pharmacol. 2019;176:337–354. doi: 10.1111/bph.14530. 30381825 PMC6329627

[13] Robb CT, Goepp M, Rossi AG, Yao C. Non-steroidal anti-inflammatory drugs, prostaglandins, and COVID-19. Br J Pharmacol. 2020;177:4899–4920. doi: 10.1111/bph.15206. 32700336 PMC7405053

[14] Roussel C, Lessard-Lord J, Nallabelli N, Muller C, Flamand N, Silvestri C, Di Marzo V. Human gut microbes produce EPA- and DHA-Derived oxylipins, but not N-Acyl-Ethanolamines, from fish oil. FASEB J. 2025;39:e70713. doi: 10.1096/fj.202500752RR. 40515551

[15] Avila-Roman J, Arreaza-Gil V, Cortes-Espinar AJ, Soliz-Rueda JR, Mulero M, Muguerza B, Arola-Arnal A, Arola L, Torres-Fuentes C. Impact of gut microbiota on plasma oxylipins profile under healthy and obesogenic conditions. Clin Nutr. 2021;40:1475–1486. doi: 10.1016/j.clnu.2021.02.035. 33743282

[16] Goepp M, Milburn JV, Zhang B, Dong Y, Tyrrell V, Zheng X, Marshall JM, Bolsega S, Basic M, Glendinning L, et al. Age-related impairment of intestinal inflammation resolution through an eicosanoid-immune-microbiota axis. Cell Host Microbe. 2025;33:671–687 e6. doi: 10.1016/j.chom.2025.04.014. 40373750

[17] Crittenden S, Goepp M, Pollock J, Robb CT, Smyth DJ, Zhou Y, Andrews R, Tyrrell V, Gkikas K, Adima A, et al. Prostaglandin E(2) promotes intestinal inflammation via inhibiting microbiota-dependent regulatory T cells. Sci Adv. 2021;7. doi: 10.1126/sciadv.abd7954. PMC7880593 33579710

[18] Sato H, Taketomi Y, Murase R, Park J, Hosomi K, Sanada TJ, Mizuguchi K, Arita M, Kunisawa J, Murakami M. Group X phospholipase A(2) links colonic lipid homeostasis to systemic metabolism via host-microbiota interaction. Cell Rep. 2024;43:114752. doi: 10.1016/j.celrep.2024.114752. 39298315

[19] Kindt A, Liebisch G, Clavel T, Haller D, Hormannsperger G, Yoon H, Kolmeder D, Sigruener A, Krautbauer S, Seeliger C, et al. The gut microbiota promotes hepatic fatty acid desaturation and elongation in mice. Nat Commun. 2018;9:3760. doi: 10.1038/s41467-018-05767-4. 30218046 PMC6138742

[20] Jing N, Edin ML, Wang Y, Yang J, Lih FB, Yeliseyev V, Liu F, Ding Y, Zeldin DC, Zhang G. The gut microbiota mediates epoxy eicosanoid metabolism in the colon. J Biol Chem. 2025;301:110338. doi: 10.1016/j.jbc.2025.110338. 40480636 PMC12269486

[21] Brown EM, Clardy J, Xavier RJ. Gut microbiome lipid metabolism and its impact on host physiology. Cell Host Microbe. 2023;31:173–186. doi: 10.1016/j.chom.2023.01.009. 36758518 PMC10124142

[22] Wang B, Wu L, Chen J, Dong L, Chen C, Wen Z, Hu J, Fleming I, Wang DW. Metabolism pathways of arachidonic acids: mechanisms and potential therapeutic targets. Signal Transduct Target Ther. 2021;6:94. doi: 10.1038/s41392-020-00443-w. 33637672 PMC7910446

[23] Patterson E, Wall R, Fitzgerald GF, Ross RP, Stanton C. Health implications of high dietary omega-6 polyunsaturated fatty acids. J Nutr Metab. 2012;2012:539426. doi: 10.1155/2012/539426. 22570770 PMC3335257

[24] Fischer R, Konkel A, Mehling H, Blossey K, Gapelyuk A, Wessel N, von Schacky C, Dechend R, Muller DN, Rothe M, et al. Dietary omega-3 fatty acids modulate the eicosanoid profile in man primarily via the CYP-epoxygenase pathway. J Lipid Res. 2014;55:1150–1164. doi: 10.1194/jlr.M047357. 24634501 PMC4031946

[25] Miyamoto J, Igarashi M, Watanabe K, Karaki SI, Mukouyama H, Kishino S, Li X, Ichimura A, Irie J, Sugimoto Y, et al. Gut microbiota confers host resistance to obesity by metabolizing dietary polyunsaturated fatty acids. Nat Commun. 2019;10:4007. doi: 10.1038/s41467-019-11978-0. 31488836 PMC6728375

[26] Kishino S, Takeuchi M, Park SB, Hirata A, Kitamura N, Kunisawa J, Kiyono H, Iwamoto R, Isobe Y, Arita M, et al. Polyunsaturated fatty acid saturation by gut lactic acid bacteria affecting host lipid composition. Proc Natl Acad Sci U S A. 2013;110:17808–17813. doi: 10.1073/pnas.1312937110. 24127592 PMC3816446

[27] Huyan Z, Pellegrini N, Steegenga W, Capuano E. Insights into gut microbiota metabolism of dietary lipids: the case of linoleic acid. Food Funct. 2022;13:4513–4526. doi: 10.1039/d1fo04254h. 35348564

[28] Devillard E, McIntosh FM, Duncan SH, Wallace RJ. Metabolism of linoleic acid by human gut bacteria: different routes for biosynthesis of conjugated linoleic acid. J Bacteriol. 2007;189:2566–2570. doi: 10.1128/JB.01359-06. 17209019 PMC1899373

[29] Pujo J, Petitfils C, Le Faouder P, Eeckhaut V, Payros G, Maurel S, Perez-Berezo T, Van Hul M, Barreau F, Blanpied C, et al. Bacteria-derived long chain fatty acid exhibits anti-inflammatory properties in colitis. Gut. 2021;70:1088–1097. doi: 10.1136/gutjnl-2020-321173. 32978245

[30] Semova I, Carten JD, Stombaugh J, Mackey LC, Knight R, Farber SA, Rawls JF. Microbiota regulate intestinal absorption and metabolism of fatty acids in the zebrafish. Cell Host Microbe. 2012;12:277–288. doi: 10.1016/j.chom.2012.08.003. 22980325 PMC3517662

[31] Weninger SN, Herman C, Meyer RK, Beauchemin ET, Kangath A, Lane AI, Martinez TM, Hasneen T, Jaramillo SA, Lindsey J, et al. Oligofructose improves small intestinal lipid-sensing mechanisms via alterations to the small intestinal microbiota. Microbiome. 2023;11:169. doi: 10.1186/s40168-023-01590-2. 37533066 PMC10394784

[32] Wang Y, Wang M, Chen J, Li Y, Kuang Z, Dende C, Raj P, Quinn G, Hu Z, Srinivasan T, et al. The gut microbiota reprograms intestinal lipid metabolism through long noncoding RNA Snhg9. Science. 2023;381:851–857. doi: 10.1126/science.ade0522. 37616368 PMC10688608

[33] Taketomi Y, Miki Y, Murakami M. Old but new: group IIA phospholipase A(2) as a modulator of gut microbiota. Metabolites. 2022;12:352. doi: 10.3390/metabo12040352. 35448539 PMC9029192

[34] Zhang G, Sun Q, Sun J, Jing N, Edin M, Wang Y, Xu W, Zhang J, Wang W, Lih F, et al. Gut microbiota protects against liver injury and fibrosis via activation of the CYP eicosanoid pathway. Res Sq. 2025. doi: 10.21203/rs.3.rs-6356145/v1.

[35] Sheppe AEF, Edelmann MJ. Roles of eicosanoids in regulating inflammation and neutrophil migration as an innate host response to bacterial infections. Infect Immun. 2021;89:e0009521. doi: 10.1128/IAI.00095-21. 34031130 PMC8281227

[36] Kim H, Rhee SH, Kokkotou E, Na X, Savidge T, Moyer MP, Pothoulakis C, LaMont JT. Clostridium difficile toxin A regulates inducible cyclooxygenase-2 and prostaglandin E2 synthesis in colonocytes via reactive oxygen species and activation of p38 MAPK. J Biol Chem. 2005;280:21237–21245. doi: 10.1074/jbc.M413842200. 15767259

[37] Uribe G, Villeger R, Bressollier P, Dillard RN, Worthley DL, Wang TC, Powell DW, Urdaci MC, Pinchuk IV. Lactobacillus rhamnosus GG increases cyclooxygenase-2 expression and prostaglandin E2 secretion in colonic myofibroblasts via a MyD88-dependent mechanism during homeostasis. Cell Microbiol. 2018;20:e12871. doi: 10.1111/cmi.12871. 29920917 PMC6202218

[38] Dennis EA, Norris PC. Eicosanoid storm in infection and inflammation. Nat Rev Immunol. 2015;15:511–523. doi: 10.1038/nri3859. 26139350 PMC4606863

[39] Miek L, Jordan PM, Gunther K, Pace S, Beyer T, Kowalak D, Hoerr V, Loffler B, Tuchscherr L, Serhan CN, et al. Staphylococcus aureus controls eicosanoid and specialized pro-resolving mediator production via lipoteichoic acid. Immunology. 2022;166:47–67. doi: 10.1111/imm.13449. 35143048 PMC9426618

[40] Jang AY, Rod-In W, Monmai C, Sohn M, Kim TR, Jeon MG, Park WJ. Anti-inflammatory potential of lactobacillus reuteri LM1071 via eicosanoid regulation in LPS-stimulated RAW264.7 cells. J Appl Microbiol. 2022;133:67–75. doi: 10.1111/jam.15331. 34688224

[41] Mukhopadhya I, Louis P. Gut microbiota-derived short-chain fatty acids and their role in human health and disease. Nat Rev Microbiol. 2025;23:635–651. doi: 10.1038/s41579-025-01183-w. 40360779

[42] Furusawa Y, Obata Y, Fukuda S, Endo TA, Nakato G, Takahashi D, Nakanishi Y, Uetake C, Kato K, Kato T, et al. Commensal microbe-derived butyrate induces the differentiation of colonic regulatory T cells. Nature. 2013;504:446–450. doi: 10.1038/nature12721. 24226770

[43] Arpaia N, Campbell C, Fan X, Dikiy S, van der Veeken J, deRoos P, Liu H, Cross JR, Pfeffer K, Coffer PJ, et al. Metabolites produced by commensal bacteria promote peripheral regulatory T-cell generation. Nature. 2013;504:451–455. doi: 10.1038/nature12726. 24226773 PMC3869884

[44] Smith PM, Howitt MR, Panikov N, Michaud M, Gallini CA, Bohlooly YM, Glickman JN, Garrett WS. The microbial metabolites, short-chain fatty acids, regulate colonic treg cell homeostasis. Science. 2013;341:569–573. doi: 10.1126/science.1241165. 23828891 PMC3807819

[45] Larraufie P, Martin-Gallausiaux C, Lapaque N, Dore J, Gribble FM, Reimann F, Blottiere HM. SCFAs strongly stimulate PYY production in human enteroendocrine cells. Sci Rep. 2018;8:74. doi: 10.1038/s41598-017-18259-0. 29311617 PMC5758799

[46] Fukuda S, Toh H, Hase K, Oshima K, Nakanishi Y, Yoshimura K, Tobe T, Clarke JM, Topping DL, Suzuki T, et al. Bifidobacteria can protect from enteropathogenic infection through production of acetate. Nature. 2011;469:543–547. doi: 10.1038/nature09646. 21270894

[47] Willemsen LE, Koetsier MA, van Deventer SJ, van Tol EA. Short chain fatty acids stimulate epithelial mucin 2 expression through differential effects on prostaglandin E(1) and E(2) production by intestinal myofibroblasts. Gut. 2003;52:1442–1447. doi: 10.1136/gut.52.10.1442. 12970137 PMC1773837

[48] Cox MA, Jackson J, Stanton M, Rojas-Triana A, Bober L, Laverty M, Yang X, Zhu F, Liu J, Wang S, et al. Short-chain fatty acids act as antiinflammatory mediators by regulating prostaglandin E(2) and cytokines. World J Gastroenterol. 2009;15:5549–5557. doi: 10.3748/wjg.15.5549. 19938193 PMC2785057

[49] Kovarik JJ, Holzl MA, Hofer J, Waidhofer-Sollner P, Sobanov Y, Koeffel R, Saemann MD, Mechtcheriakova D, Zlabinger GJ. Eicosanoid modulation by the short-chain fatty acid n-butyrate in human monocytes. Immunology. 2013;139:395–405. doi: 10.1111/imm.12089. 23398566 PMC3701186

[50] Fork C, Vasconez AE, Janetzko P, Angioni C, Schreiber Y, Ferreiros N, Geisslinger G, Leisegang MS, Steinhilber D, Brandes RP. Epigenetic control of microsomal prostaglandin E synthase-1 by HDAC-mediated recruitment of p300. J Lipid Res. 2017;58:386–392. doi: 10.1194/jlr.M072280. 27913583 PMC5282954

[51] Tong X, Yin L, Giardina C. Butyrate suppresses Cox-2 activation in colon cancer cells through HDAC inhibition. Biochem Biophys Res Commun. 2004;317:463–471. doi: 10.1016/j.bbrc.2004.03.066. 15063780

[52] Kim MH, Kang SG, Park JH, Yanagisawa M, Kim CH. Short-chain fatty acids activate GPR41 and GPR43 on intestinal epithelial cells to promote inflammatory responses in mice. Gastroenterology. 2013;145:396–406 e1-10. doi: 10.1053/j.gastro.2013.04.056. 23665276

[53] Ashton JS, Roberts JW, Wakefield CJ, Page RM, MacLaren DPM, Marwood S, Malone JJ. The effects of medium chain triglyceride (MCT) supplementation using a C(8):C(10) ratio of 30:70 on cognitive performance in healthy young adults. Physiol Behav. 2021;229:113252. doi: 10.1016/j.physbeh.2020.113252. 33220329

[54] Shcherbakova K, Schwarz A, Ivleva I, Nikitina V, Krytskaya D, Apryatin S, Karpenko M, Trofimov A. Short- and long-term cognitive and metabolic effects of medium-chain triglyceride supplementation in rats. Heliyon. 2023;9:e13446. doi: 10.1016/j.heliyon.2023.e13446. 36825166 PMC9941952

[55] Wang ME, Singh BK, Hsu MC, Huang C, Yen PM, Wu LS, Jong DS, Chiu CH. Increasing dietary medium-chain fatty acid ratio mitigates high-fat diet-induced non-alcoholic steatohepatitis by regulating autophagy. Sci Rep. 2017;7:13999. doi: 10.1038/s41598-017-14376-y. 29070903 PMC5656678

[56] Lee SI, Kang KS. Function of capric acid in cyclophosphamide-induced intestinal inflammation, oxidative stress, and barrier function in pigs. Sci Rep. 2017;7:16530. doi: 10.1038/s41598-017-16561-5. 29184078 PMC5705592

[57] Xu C, Liu Q, Xu M, Ayalew H, Iqbal W, Lin J, Song J, Jin L, Song Z, Zhang H. Dietary medium-chain fatty acids mitigate escherichia coli O78 infection in broilers by enhancing intestinal immune barrier and modulating microbiota. Poult Sci. 2025;104:105775. doi: 10.1016/j.psj.2025.105775. 40961776 PMC12475841

[58] Rani S, Verma S, Singh H, Ram C. Antibacterial activity and mechanism of essential oils in combination with medium-chain fatty acids against predominant bovine mastitis pathogens. Lett Appl Microbiol. 2022;74:959–969. doi: 10.1111/lam.13675. 35178733

[59] Yen HC, Lai WK, Lin CS, Chiang SH. Medium-chain triglyceride as an alternative of in-feed colistin sulfate to improve growth performance and intestinal microbial environment in newly weaned pigs. Anim Sci J. 2015;86:99–104. doi: 10.1111/asj.12248. 25039368

[60] De Preter V, Machiels K, Joossens M, Arijs I, Matthys C, Vermeire S, Rutgeerts P, Verbeke K. Faecal metabolite profiling identifies medium-chain fatty acids as discriminating compounds in IBD. Gut. 2015;64:447–458. doi: 10.1136/gutjnl-2013-306423. 24811995

[61] Saresella M, Marventano I, Barone M, La Rosa F, Piancone F, Mendozzi L, d'Arma A, Rossi V, Pugnetti L, Roda G, et al. Alterations in circulating fatty acid are associated with gut microbiota dysbiosis and inflammation in multiple sclerosis. Front Immunol. 2020;11:1390. doi: 10.3389/fimmu.2020.01390. 32733460 PMC7358580

[62] Cox TO, Devason AS, de Araujo A, Mason S, Subramanian M, Salvador AFM, Descamps HC, Kim J, Zhu Y, Litichevskiy L, et al. Intestinal interoceptive dysfunction drives age-associated cognitive decline. Nature. 2026;652:442–450. doi: 10.1038/s41586-026-10191-6. 41813891 PMC13061634

[63] Deleu S, Machiels K, Raes J, Verbeke K, Vermeire S. Short chain fatty acids and its producing organisms: an overlooked therapy for IBD? EBioMedicine. 2021;66:103293. doi: 10.1016/j.ebiom.2021.103293. 33813134 PMC8047503

[64] Litvak Y, Byndloss MX, Tsolis RM, Baumler AJ. Dysbiotic proteobacteria expansion: a microbial signature of epithelial dysfunction. Curr Opin Microbiol. 2017;39:1–6. doi: 10.1016/j.mib.2017.07.003. 28783509

[65] Santana PT, Rosas SLB, Ribeiro BE, Marinho Y, de Souza HSP. Dysbiosis in inflammatory bowel disease: pathogenic role and potential therapeutic targets. Int J Mol Sci. 2022;23:3464. doi: 10.3390/ijms23073464. 35408838 PMC8998182

[66] Needham BD, Trent MS. Fortifying the barrier: the impact of lipid A remodelling on bacterial pathogenesis. Nat Rev Microbiol. 2013;11:467–481. doi: 10.1038/nrmicro3047. 23748343 PMC6913092

[67] Harberts EM, Grubaugh D, Akuma DC, Shin S, Ernst RK, Brodsky IE. Position-specific secondary acylation determines detection of lipid A by murine TLR4 and Caspase-11. Infect Immun. 2022;90:e0020122. doi: 10.1128/iai.00201-22. 35862717 PMC9387250

[68] McDonnell M, Liang Y, Noronha A, Coukos J, Kasper DL, Farraye FA, Ganley-Leal LM. Systemic toll-like receptor ligands modify B-cell responses in human inflammatory bowel disease. Inflamm Bowel Dis. 2011;17:298–307. doi: 10.1002/ibd.21424. 20806343

[69] Qi HY, Shelhamer JH. Toll-like receptor 4 signaling regulates cytosolic phospholipase A2 activation and lipid generation in lipopolysaccharide-stimulated macrophages. J Biol Chem. 2005;280:38969–38975. doi: 10.1074/jbc.M509352200. 16176925

[70] Schaldach CM, Riby J, Bjeldanes LF. Lipoxin A4: a new class of ligand for the ah receptor. Biochemistry. 1999;38:7594–7600. doi: 10.1021/bi982861e. 10360957

[71] Nebert DW, Karp CL. Endogenous functions of the aryl hydrocarbon receptor (AHR): intersection of cytochrome P450 1 (CYP1)-metabolized eicosanoids and AHR biology. J Biol Chem. 2008;283:36061–36065. doi: 10.1074/jbc.R800053200. 18713746 PMC2606007

[72] Marion-Letellier R, Savoye G, Ghosh S. Fatty acids, eicosanoids and PPAR gamma. Eur J Pharmacol. 2016;785:44–49. doi: 10.1016/j.ejphar.2015.11.004. 26632493

[73] Evans RJ, Pline K, Loynes CA, Needs S, Aldrovandi M, Tiefenbach J, Bielska E, Rubino RE, Nicol CJ, May RC, et al. 15-keto-prostaglandin E2 activates host peroxisome proliferator-activated receptor gamma (PPAR-gamma) to promote cryptococcus neoformans growth during infection. PLoS Pathog. 2019;15:e1007597. doi: 10.1371/journal.ppat.1007597. 30921435 PMC6438442

[74] Matsumoto Y, Nakanishi Y, Yoshioka T, Yamaga Y, Masuda T, Fukunaga Y, Sono M, Yoshikawa T, Nagao M, Araki O, et al. Epithelial EP4 plays an essential role in maintaining homeostasis in colon. Sci Rep. 2019;9:15244. doi: 10.1038/s41598-019-51639-2. 31645712 PMC6811535

[75] Na YR, Jung D, Stakenborg M, Jang H, Gu GJ, Jeong MR, Suh SY, Kim HJ, Kwon YH, Sung TS, et al. Prostaglandin E(2) receptor PTGER4-expressing macrophages promote intestinal epithelial barrier regeneration upon inflammation. Gut. 2021;70:2249–2260. doi: 10.1136/gutjnl-2020-322146. 33558271

[76] Zackular JP, Kirk L, Trindade BC, Skaar EP, Aronoff DM. Misoprostol protects mice against severe clostridium difficile infection and promotes recovery of the gut microbiota after antibiotic perturbation. Anaerobe. 2019;58:89–94. doi: 10.1016/j.anaerobe.2019.06.006. 31220605 PMC6697607

[77] Duffin R, O'Connor RA, Crittenden S, Forster T, Yu C, Zheng X, Smyth D, Robb CT, Rossi F, Skouras C, et al. Prostaglandin E(2) constrains systemic inflammation through an innate lymphoid cell-IL-22 axis. Science. 2016;351:1333–1338. doi: 10.1126/science.aad9903. 26989254 PMC4841390

[78] Rodriguez-Lagunas MJ, Martin-Venegas R, Moreno JJ, Ferrer R. PGE2 promotes Ca2+-mediated epithelial barrier disruption through EP1 and EP4 receptors in Caco-2 cell monolayers. Am J Physiol Cell Physiol. 2010;299:C324–34. doi: 10.1152/ajpcell.00397.2009. 20484658

[79] Dong Y, Johnson BA, Ruan L, Zeineldin M, Bi T, Liu AZ, Raychaudhuri S, Chiu I, Zhu J, Smith B, et al. Disruption of epithelium integrity by inflammation-associated fibroblasts through prostaglandin signaling. Sci Adv. 2024;10:eadj7666. doi: 10.1126/sciadv.adj7666. 38569041 PMC10990275

[80] Lejeune M, Leung P, Beck PL, Chadee K. Role of EP4 receptor and prostaglandin transporter in prostaglandin E2-induced alteration in colonic epithelial barrier integrity. Am J Physiol Gastrointest Liver Physiol. 2010;299:G1097–105. doi: 10.1152/ajpgi.00280.2010. 20813914

[81] Khan KN, Kitajima M, Yamaguchi N, Fujishita A, Nakashima M, Ishimaru T, Masuzaki H. Role of prostaglandin E2 in bacterial growth in women with endometriosis. Hum Reprod. 2012;27:3417–3424. doi: 10.1093/humrep/des331. 23001777

[82] Cai JY, Hou YN, Li J, Ma K, Yao GD, Liu WW, Hayashi T, Itoh K, Tashiro SI, Onodera S, et al. Prostaglandin E2 attenuates synergistic bactericidal effects between COX inhibitors and antibiotics on staphylococcus aureus. Prostaglandins Leukot Essent Fatty Acids. 2018;133:16–22. doi: 10.1016/j.plefa.2018.04.005. 29789128

[83] Pinchaud K, Hafeez Z, Auger S, Chatel JM, Chadi S, Langella P, Paoli J, Dary-Mourot A, Maguin-Gate K, Olivier JL. Impact of dietary arachidonic acid on gut microbiota composition and gut-brain axis in Male BALB/C mice. Nutrients. 2022;14:5338. doi: 10.3390/nu14245338. 36558497 PMC9786182

[84] Rehman A, Pham V, Seifert N, Richard N, Sybesma W, Steinert RE. The polyunsaturated fatty acids eicosapentaenoic acid and docosahexaenoic acid, and vitamin K(1) modulate the gut microbiome: a study using an in vitro shime model. J Diet Suppl. 2024;21:135–153. doi: 10.1080/19390211.2023.2198007. 37078491

[85] Aldoori J, Mitra S, Davie A, Toogood GJ, Edwards C, Hull MA. The effect of omega-3 polyunsaturated fatty acids on short-chain fatty acid production and the gut microbiome in an in vitro colonic fermentation model. Gut Microbiome (Camb). 2026;7:e1. doi: 10.1017/gmb.2025.10016. 41608298 PMC12835959

[86] Chai Z, Zhang H, Ji X, Hu X, He Y, Zhao F, Song C, Zhou Y, Li T, He C, et al. The disparate effects of omega-3 PUFAs on intestinal microbial homeostasis in experimental rodents under physiological condition. Prostaglandins Leukot Essent Fatty Acids. 2024;203:102643. doi: 10.1016/j.plefa.2024.102643. 39317024

[87] Altendorfer B, Benedetti A, Mrowetz H, Bernegger S, Bretl A, Preishuber-Pflugl J, Bessa de Sousa DM, Ladek AM, Koller A, Le Faouder P, et al. Omega-3 EPA supplementation shapes the gut microbiota composition and reduces major histocompatibility complex class II in aged wild-type and APP/PS1 Alzheimer's mice: a pilot experimental study. Nutrients. 2025;17:1108. doi: 10.3390/nu17071108. 40218866 PMC11990804

[88] Fang J, Zhang Z, Cheng Y, Yang H, Zhang H, Xue Z, Lu S, Dong Y, Song C, Zhang X, et al. EPA and DHA differentially coordinate the crosstalk between host and gut microbiota and block DSS-induced colitis in mice by a reinforced colonic mucus barrier. Food Funct. 2022;13:4399–4420. doi: 10.1039/d1fo03815j. 35297435

[89] Dong Y, Huang C, Yang J, Zheng Z, Dai Z. Docosapentaenoic acid (DPA, 22:5n-3) alleviates ulcerative colitis via modification of gut microbiota and their metabolism. Nutrients. 2022;14:4204. doi: 10.3390/nu14194204. 36235856 PMC9570819

[90] Liang X, Bittinger K, Li X, Abernethy DR, Bushman FD, FitzGerald GA. Bidirectional interactions between indomethacin and the murine intestinal microbiota. Elife. 2015;4:e08973. doi: 10.7554/eLife.08973. 26701907 PMC4755745

[91] Xiao X, Nakatsu G, Jin Y, Wong S, Yu J, Lau JY. Gut microbiota mediates protection against enteropathy induced by indomethacin. Sci Rep. 2017;7:40317. doi: 10.1038/srep40317. 28067296 PMC5220306

[92] Haghighi A, Toth AS, Demeter ZO, Hutka B, Zsidai A, Lengyel L, Haghighi S, Pannier M, Le Cosquer G, Meunier ES, et al. Oral indomethacin modifies small intestine biofilms and host-microbe interaction mediators. Life Sci. 2026;384:124114. doi: 10.1016/j.lfs.2025.124114. 41314593

[93] Maseda D, Zackular JP, Trindade B, Kirk L, Roxas JL, Rogers LM, Washington MK, Du L, Koyama T, Viswanathan VK, et al. Nonsteroidal anti-inflammatory drugs alter the microbiota and exacerbate clostridium difficile colitis while dysregulating the inflammatory response. mBio. 2019;10. doi: 10.1128/mBio.02282-18. PMC6325247 30622186

[94] Hutka B, Lazar B, Toth AS, Agg B, Laszlo SB, Makra N, Ligeti B, Scheich B, Kiraly K, Al-Khrasani M, et al. The nonsteroidal anti-inflammatory drug ketorolac alters the small intestinal microbiota and bile acids without inducing intestinal damage or delaying peristalsis in the rat. Front Pharmacol. 2021;12:664177. doi: 10.3389/fphar.2021.664177. 34149417 PMC8213092

[95] Rogers MAM, Aronoff DM. The influence of non-steroidal anti-inflammatory drugs on the gut microbiome. Clin Microbiol Infect. 2016;22:178.e1–178.e9. doi: 10.1016/j.cmi.2015.10.003. PMC4754147 26482265

[96] Prizment AE, Staley C, Onyeaghala GC, Vivek S, Thyagarajan B, Straka RJ, Demmer RT, Knights D, Meyer KA, Shaukat A, et al. Randomised clinical study: oral aspirin 325 mg daily vs placebo alters gut microbial composition and bacterial taxa associated with colorectal cancer risk. Aliment Pharmacol Ther. 2020;52:976–987. doi: 10.1111/apt.16013. 32770859 PMC7719064

[97] Hopke A, Lin T, Scherer AK, Shay AE, Timmer KD, Wilson-Mifsud B, Mansour MK, Serhan CN, Irimia D, Hurley BP. Transcellular biosynthesis of leukotriene B(4) orchestrates neutrophil swarming to fungi. iScience. 2022;25:105226. doi: 10.1016/j.isci.2022.105226. 36267914 PMC9576560

[98] Kim ND, Chou RC, Seung E, Tager AM, Luster AD. A unique requirement for the leukotriene B4 receptor BLT1 for neutrophil recruitment in inflammatory arthritis. J Exp Med. 2006;203:829–835. doi: 10.1084/jem.20052349. 16567386 PMC2118298

[99] Lee M, Boyce JA, Barrett NA. Cysteinyl leukotrienes in allergic inflammation. Annu Rev Pathol. 2025;20:115–141. doi: 10.1146/annurev-pathmechdis-111523-023509. 39374430 PMC11759657

[100] Yonetomi Y, Sekioka T, Kadode M, Kitamine T, Kamiya A, Matsumura N, Fujita M, Kawabata K. Leukotriene C4 induces bronchoconstriction and airway vascular hyperpermeability via the cysteinyl leukotriene receptor 2 in S-hexyl glutathione-treated Guinea pigs. Eur J Pharmacol. 2015;754:98–104. doi: 10.1016/j.ejphar.2015.02.014. 25704617

[101] Jala VR, Maturu P, Bodduluri SR, Krishnan E, Mathis S, Subbarao K, Wang M, Jenson AB, Proctor ML, Rouchka EC, et al. Leukotriene B(4)-receptor-1 mediated host response shapes gut microbiota and controls colon tumor progression. Oncoimmunology. 2017;6:e1361593. doi: 10.1080/2162402X.2017.1361593. 29209564 PMC5706601

[102] Hayashi A, Sakamoto N, Kobayashi K, Murata T. Enhancement of prostaglandin D(2)-D prostanoid 1 signaling reduces intestinal permeability by stimulating mucus secretion. Front Immunol. 2023;14:1276852. doi: 10.3389/fimmu.2023.1276852. 37942331 PMC10628818

[103] Bessard A, Cardaillac C, Oullier T, Cenac N, Rolli-Derkinderen M, Neunlist M, Venara A. Alterations of prostanoid expression and intestinal epithelial barrier functions in ileus. J Surg Res. 2024;296:165–173. doi: 10.1016/j.jss.2023.12.018. 38277953

[104] Pochard C, Gonzales J, Bessard A, Mahe MM, Bourreille A, Cenac N, Jarry A, Coron E, Podevin J, Meurette G, et al. PGI(2) inhibits intestinal epithelial permeability and apoptosis to alleviate colitis. Cell Mol Gastroenterol Hepatol. 2021;12:1037–1060. doi: 10.1016/j.jcmgh.2021.05.001. 33971327 PMC8342971

[105] Rodriguez-Lagunas MJ, Storniolo CE, Ferrer R, Moreno JJ. 5-Hydroxyeicosatetraenoic acid and leukotriene D4 increase intestinal epithelial paracellular permeability. Int J Biochem Cell Biol. 2013;45:1318–1326. doi: 10.1016/j.biocel.2013.04.005. 23583294

[106] Cabrera G, Fernandez-Brando RJ, Mejias MP, Ramos MV, Abrey-Recalde MJ, Vanzulli S, Vermeulen M, Palermo MS. Leukotriene C4 increases the susceptibility of adult mice to shiga toxin-producing escherichia coli infection. Int J Med Microbiol. 2015;305:910–917. doi: 10.1016/j.ijmm.2015.09.006. 26456732

[107] Robb CT, McSorley HJ, Lee J, Aoki T, Yu C, Crittenden S, Astier A, Felton JM, Parkinson N, Ayele A, et al. Prostaglandin E(2) stimulates adaptive IL-22 production and promotes allergic contact dermatitis. J Allergy Clin Immunol. 2018;141:152–162. doi: 10.1016/j.jaci.2017.04.045. 28583370 PMC5626002

[108] Wang PC, Liu ZK, Li JR, Zhao ZH, Chang QW, Guo XM, Jin L, Hu YT, Yang Z. Tryptophan regulates the expression of IGFBP1 in bovine endometrial epithelial cells in vitro via the TDO2-AHR pathway. BMC Vet Res. 2024;20:390. doi: 10.1186/s12917-024-04191-9. 39227948 PMC11373120

[109] Wahli W, Michalik L. PPARs at the crossroads of lipid signaling and inflammation. Trends Endocrinol Metab. 2012;23:351–363. doi: 10.1016/j.tem.2012.05.001. 22704720

[110] Meyer M, Grabherr F, Plattner C, Hadjihannas MV, Rao Z, Marteau V, Lopez-Agudelo VA, Schwarzler J, Mayr L, Jukic A, et al. TE. Metabolic stress sensing by epithelial RXRalpha links westernization of diet with Crohn's disease. Cell Metab. 2025. doi: 10.1016/j.cmet.2025.11.008. PMC13034575 41365295

[111] Kimura I, Ichimura A, Ohue-Kitano R, Igarashi M. Free fatty acid receptors in health and disease. Physiol Rev. 2020;100:171–210. doi: 10.1152/physrev.00041.2018. 31487233

[112] Smyth M, Lunken G, Jacobson K. Insights into inflammatory bowel disease and effects of dietary fatty acid intake with a focus on polyunsaturated fatty acids using preclinical models. J Can Assoc Gastroenterol. 2024;7:104–114. doi: 10.1093/jcag/gwad058. 38314173 PMC10837003

[113] Rehal S, von der Weid PY. Experimental ileitis alters prostaglandin biosynthesis in mesenteric lymphatic and blood vessels. Prostaglandins Other Lipid Mediat. 2015;116-117:37–48. doi: 10.1016/j.prostaglandins.2014.11.001. 25526689

[114] Kikut J, Mokrzycka M, Drozd A, Grzybowska-Chlebowczyk U, Zietek M, Szczuko M. Involvement of proinflammatory arachidonic acid (ARA) derivatives in Crohn's disease (CD) and ulcerative colitis (UC). J Clin Med. 2022;11:1861. doi: 10.3390/jcm11071861. 35407469 PMC8999554

[115] Nemeth K, Leelahavanichkul A, Yuen PS, Mayer B, Parmelee A, Doi K, Robey PG, Leelahavanichkul K, Koller BH, Brown JM, et al. Bone marrow stromal cells attenuate sepsis via prostaglandin E(2)-dependent reprogramming of host macrophages to increase their interleukin-10 production. Nat Med. 2009;15:42–49. doi: 10.1038/nm.1905. 19098906 PMC2706487

[116] Jana B, Andronowska A, Calka J, Mowinska A. Biosynthetic pathway for leukotrienes is stimulated by lipopolysaccharide and cytokines in pig endometrial stromal cells. Sci Rep. 2025;15:2806. doi: 10.1038/s41598-025-86787-1. 39843578 PMC11754892

[117] Jiang GL, Nieves A, Im WB, Old DW, Dinh DT, Wheeler L. The prevention of colitis by E prostanoid receptor 4 agonist through enhancement of epithelium survival and regeneration. J Pharmacol Exp Ther. 2007;320:22–28. doi: 10.1124/jpet.106.111146. 17008451

[118] Patankar JV, Muller TM, Kantham S, Acera MG, Mascia F, Scheibe K, Mahapatro M, Heichler C, Yu Y, Li W, et al. E-type prostanoid receptor 4 drives resolution of intestinal inflammation by blocking epithelial necroptosis. Nat Cell Biol. 2021;23:796–807. doi: 10.1038/s41556-021-00708-8. 34239062

[119] Miyoshi H, VanDussen KL, Malvin NP, Ryu SH, Wang Y, Sonnek NM, Lai CW, Stappenbeck TS. Prostaglandin E2 promotes intestinal repair through an adaptive cellular response of the epithelium. EMBO J. 2017;36:5–24. doi: 10.15252/embj.201694660. 27797821 PMC5210160

[120] Hosono K, Kojo K, Narumiya S, Majima M, Ito Y. Prostaglandin E receptor EP4 stimulates lymphangiogenesis to promote mucosal healing during DSS-induced colitis. Biomed Pharmacother. 2020;128:110264. doi: 10.1016/j.biopha.2020.110264. 32447215

[121] Jiang Z, Waterbury QT, Malagola E, Fu N, Kim W, Ochiai Y, Wu F, Guha C, Shawber CJ, Yan KS, et al. Microbial-dependent recruitment of immature myeloid cells promotes intestinal regeneration. Cell Mol Gastroenterol Hepatol. 2024;17:321–346. doi: 10.1016/j.jcmgh.2023.10.007. 37898454 PMC10821484

[122] Roulis M, Nikolaou C, Kotsaki E, Kaffe E, Karagianni N, Koliaraki V, Salpea K, Ragoussis J, Aidinis V, Martini E, et al. Intestinal myofibroblast-specific Tpl2-Cox-2-PGE2 pathway links innate sensing to epithelial homeostasis. Proc Natl Acad Sci U S A. 2014;111:E4658–67. doi: 10.1073/pnas.1415762111. 25316791 PMC4217397

[123] Ohara D, Takeuchi Y, Hirota K. Type 17 immunity: novel insights into intestinal homeostasis and autoimmune pathogenesis driven by gut-primed T cells. Cell Mol Immunol. 2024;21:1183–1200. doi: 10.1038/s41423-024-01218-x. 39379604 PMC11528014

[124] Maseda D, Banerjee A, Johnson EM, Washington MK, Kim H, Lau KS, Crofford LJ. mPGES-1-Mediated production of PGE(2) and EP4 receptor sensing regulate T cell colonic inflammation. Front Immunol. 2018;9:2954. doi: 10.3389/fimmu.2018.02954. 30619314 PMC6302013

[125] Kabashima K, Saji T, Murata T, Nagamachi M, Matsuoka T, Segi E, Tsuboi K, Sugimoto Y, Kobayashi T, Miyachi Y, et al. The prostaglandin receptor EP4 suppresses colitis, mucosal damage and CD4 cell activation in the gut. J Clin Invest. 2002;109:883–893. doi: 10.1172/JCI14459. 11927615 PMC150928

[126] Nakatsuji M, Minami M, Seno H, Yasui M, Komekado H, Higuchi S, Fujikawa R, Nakanishi Y, Fukuda A, Kawada K, et al. EP4 receptor-associated protein in macrophages ameliorates colitis and colitis-associated tumorigenesis. PLoS Genet. 2015;11:e1005542. doi: 10.1371/journal.pgen.1005542. 26439841 PMC4595503

[127] Murray P, Byunghyun Kang EE, Kelsall BL. Single-cell mRNA analysis of colon tissue during DSS colitis in CX3CR1cre/+EP4fl/fl mice suggests PGE2 signaling via EP4 promotes survival of protective macrophages. The Journal of Immunology. 2022;208:171.10–171.10. doi: 10.4049/jimmunol.208.Supp.171.10.

[128] Murray P. BK. Prostaglandin E2 signaling via its receptor EP4 on macrophages protects against acute colitis by preserving intestinal barrier function. The Journal of Immunology. 2023;210:61.15–61.15. doi: 10.4049/jimmunol.210.Supp.61.15.36445376

[129] Yao C, Hirata T, Soontrapa K, Ma X, Takemori H, Narumiya S. Prostaglandin E(2) promotes Th1 differentiation via synergistic amplification of IL-12 signalling by cAMP and PI3-kinase. Nat Commun. 2013;4:1685. doi: 10.1038/ncomms2684. 23575689 PMC3644078

[130] Goepp M, Crittenden S, Zhou Y, Rossi AG, Narumiya S, Yao C. Prostaglandin E(2) directly inhibits the conversion of inducible regulatory T cells through EP2 and EP4 receptors via antagonizing TGF-beta signalling. Immunology. 2021;164:777–791. doi: 10.1111/imm.13417. 34529833 PMC8561111

[131] Wang C, Yu T, Wang Y, Xu M, Wang J, Zhao Y, Wan Q, Wang L, Yang J, Zhou J, et al. Targeting the EP2 receptor ameliorates inflammatory bowel disease in mice by enhancing the immunosuppressive activity of T(reg) cells. Mucosal Immunol. 2025;18:418–430. doi: 10.1016/j.mucimm.2024.12.014. 39746548

[132] Thumkeo D, Punyawatthananukool S, Prasongtanakij S, Matsuura R, Arima K, Nie H, Yamamoto R, Aoyama N, Hamaguchi H, Sugahara S, et al. PGE(2)-EP2/EP4 signaling elicits immunosuppression by driving the mregDC-Treg axis in inflammatory tumor microenvironment. Cell Rep. 2022;39:110914. doi: 10.1016/j.celrep.2022.110914. 35675777

[133] Matsuura R, Punyawatthananukool S, Kawakami R, Mikami N, Sakaguchi S, Narumiya S. Prostaglandin E(2)-EP2/EP4 signaling induces the tumor-infiltrating treg phenotype for tumor growth. Proc Natl Acad Sci U S A. 2025;122:e2424251122. doi: 10.1073/pnas.2424251122. 41343674 PMC12704795

[134] Yao C, Sakata D, Esaki Y, Li Y, Matsuoka T, Kuroiwa K, Sugimoto Y, Narumiya S. Prostaglandin E2-EP4 signaling promotes immune inflammation through Th1 cell differentiation and Th17 cell expansion. Nat Med. 2009;15:633–640. doi: 10.1038/nm.1968. 19465928

[135] Jostins L, Ripke S, Weersma RK, Duerr RH, McGovern DP, Hui KY, Lee JC, Schumm LP, Sharma Y, Anderson CA, et al. Cho JH. Host-microbe interactions have shaped the genetic architecture of inflammatory bowel disease. Nature. 2012;491:119–124. doi: 10.1038/nature11582. 23128233 PMC3491803

[136] Li Y, Soendergaard C, Bergenheim FH, Aronoff DM, Milne G, Riis LB, Seidelin JB, Jensen KB, Nielsen OH. COX-2-PGE(2) signaling impairs intestinal epithelial regeneration and associates with TNF inhibitor responsiveness in ulcerative colitis. EBioMedicine. 2018;36:497–507. doi: 10.1016/j.ebiom.2018.08.040. 30190207 PMC6197735

[137] Mukhopadhyay S, Heinz E, Porreca I, Alasoo K, Yeung A, Yang HT, Schwerd T, Forbester JL, Hale C, Agu CA, et al. Loss of IL-10 signaling in macrophages limits bacterial killing driven by prostaglandin E2. J Exp Med. 2020;217. doi: 10.1084/jem.20180649. PMC7041704 31819956

[138] Singh VP, Patil CS, Jain NK, Singh A, Kulkarni SK. Effect of nimesulide on acetic acid- and leukotriene-induced inflammatory bowel disease in rats. Prostaglandins Other Lipid Mediat. 2003;71:163–175. doi: 10.1016/s1098-8823(03)00038-8. 14518559

[139] Li Y, Yang S, Lun J, Gao J, Gao X, Gong Z, Wan Y, He X, Cao H. Inhibitory effects of the lactobacillus rhamnosus GG effector protein HM0539 on inflammatory response through the TLR4/MyD88/NF-small ka, CyrillicB axis. Front Immunol. 2020;11:551449. doi: 10.3389/fimmu.2020.551449. 33123130 PMC7573360

[140] Yu H, Zhang S, Li R, Ma C, Zhang Q, Xia F, Zhou B, Xie Z, Liao Z. Berberine alleviates inflammation and suppresses PLA2-COX-2-PGE2-EP2 pathway through targeting gut microbiota in DSS-induced ulcerative colitis. Biochem Biophys Res Commun. 2024;695:149411. doi: 10.1016/j.bbrc.2023.149411. 38154262

[141] Jupp J, Hillier K, Elliott DH, Fine DR, Bateman AC, Johnson PA, Cazaly AM, Penrose JF, Sampson AP. Colonic expression of leukotriene-pathway enzymes in inflammatory bowel diseases. Inflamm Bowel Dis. 2007;13:537–546. doi: 10.1002/ibd.20094. 17230539

[142] Sharon P, Stenson WF. Enhanced synthesis of leukotriene B4 by colonic mucosa in inflammatory bowel disease. Gastroenterology. 1984;86:453–460. doi: 10.1016/S0016-5085(84)80015-3. 6319219

[143] Ikehata A, Hiwatashi N, Kinouchi Y, Ito K, Yamazaki H, Toyota T. Leukotriene B4 omega-hydroxylase activity in polymorphonuclear leukocytes from patients with inflammatory bowel disease. Prostaglandins Leukot Essent Fatty Acids. 1993;49:489–494. doi: 10.1016/0952-3278(93)90036-v. 8395694

[144] Rahabi M, Jacquemin G, Prat M, Meunier E, AlaEddine M, Bertrand B, Lefevre L, Benmoussa K, Batigne P, Aubouy A, et al. Divergent roles for macrophage C-type lectin receptors, Dectin-1 and mannose receptors, in the intestinal inflammatory response. Cell Rep. 2020;30:4386–4398 e5. doi: 10.1016/j.celrep.2020.03.018. 32234475

[145] Wang Y, Spatz M, Da Costa G, Michaudel C, Lapiere A, Danne C, Agus A, Michel ML, Netea MG, Langella P, et al. Deletion of both Dectin-1 and Dectin-2 affects the bacterial but not fungal gut microbiota and susceptibility to colitis in mice. Microbiome. 2022;10:91. doi: 10.1186/s40168-022-01273-4. 35698210 PMC9195441

[146] Cole AT, Pilkington BJ, McLaughlan J, Smith C, Balsitis M, Hawkey CJ. Mucosal factors inducing neutrophil movement in ulcerative colitis: the role of interleukin 8 and leukotriene B4. Gut. 1996;39:248–254. doi: 10.1136/gut.39.2.248. 8977339 PMC1383307

[147] Pazos MA, Pirzai W, Yonker LM, Morisseau C, Gronert K, Hurley BP. Distinct cellular sources of hepoxilin A3 and leukotriene B4 are used to coordinate bacterial-induced neutrophil transepithelial migration. J Immunol. 2015;194:1304–1315. doi: 10.4049/jimmunol.1402489. 25548217 PMC4297725

[148] Yokomizo T, Shimizu T. The leukotriene B(4) receptors BLT1 and BLT2 as potential therapeutic targets. Immunol Rev. 2023;317:30–41. doi: 10.1111/imr.13196. 36908237

[149] Enderes J, Mallesh S, Stein K, Wagner M, Lysson M, Schneiker B, Kalff JC, Wehner S. Treatment with the 5-Lipoxygenase antagonist zileuton protects mice from postoperative ileus. Eur Surg Res. 2022;63:224–231. doi: 10.1159/000522157. 35184063

[150] Qin J, Li Y, Cai Z, Li S, Zhu J, Zhang F, Liang S, Zhang W, Guan Y, Shen D, et al. A metagenome-wide association study of gut microbiota in type 2 diabetes. Nature. 2012;490:55–60. doi: 10.1038/nature11450. 23023125

[151] Canfora EE, Meex RCR, Venema K, Blaak EE. Gut microbial metabolites in obesity, NAFLD and T2DM. Nat Rev Endocrinol. 2019;15:261–273. doi: 10.1038/s41574-019-0156-z. 30670819

[152] Zhang L, Chu J, Hao W, Zhang J, Li H, Yang C, Yang J, Chen X, Wang H. Gut microbiota and type 2 diabetes mellitus: association, mechanism, and translational applications. Mediators Inflamm. 2021;2021:5110276. doi: 10.1155/2021/5110276. 34447287 PMC8384524

[153] Zhou Z, Sun B, Yu D, Zhu C. Gut microbiota: an important player in type 2 diabetes mellitus. Front Cell Infect Microbiol. 2022;12:834485. doi: 10.3389/fcimb.2022.834485. 35242721 PMC8886906

[154] Kolodziejczyk AA, Zheng D, Shibolet O, Elinav E. The role of the microbiome in NAFLD and NASH. EMBO Mol Med. 2019;11. doi: 10.15252/emmm.201809302. PMC6365925 30591521

[155] Boursier J, Mueller O, Barret M, Machado M, Fizanne L, Araujo-Perez F, Guy CD, Seed PC, Rawls JF, David LA, et al. The severity of nonalcoholic fatty liver disease is associated with gut dysbiosis and shift in the metabolic function of the gut microbiota. Hepatology. 2016;63:764–775. doi: 10.1002/hep.28356. 26600078 PMC4975935

[156] Tolhurst G, Heffron H, Lam YS, Parker HE, Habib AM, Diakogiannaki E, Cameron J, Grosse J, Reimann F, Gribble FM. Short-chain fatty acids stimulate glucagon-like peptide-1 secretion via the G-protein-coupled receptor FFAR2. Diabetes. 2012;61:364–371. doi: 10.2337/db11-1019. 22190648 PMC3266401

[157] Overby HB, Ferguson JF. Gut microbiota-derived short-chain fatty acids facilitate Microbiota:host cross talk and modulate obesity and hypertension. Curr Hypertens Rep. 2021;23:8. doi: 10.1007/s11906-020-01125-2. 33537923 PMC7992370

[158] Al Qassab M, Chaarani N, Hamou A, Harb R, Jradi A, Zeineddine M, Ghadieh HE, Khattar ZA, Azar S, Kanaan A, et al. The gut microbiota-insulin resistance axis: mechanisms, clinical implications, and therapeutic potential. FASEB Bioadv. 2026;8:e70080. doi: 10.1096/fba.2025-00218. 41522487 PMC12784175

[159] Aoki R, Onuki M, Hattori K, Ito M, Yamada T, Kamikado K, Kim YG, Nakamoto N, Kimura I, Clarke JM, et al. Commensal microbe-derived acetate suppresses NAFLD/NASH development via hepatic FFAR2 signalling in mice. Microbiome. 2021;9:188. doi: 10.1186/s40168-021-01125-7. 34530928 PMC8447789

[160] Miele L, Valenza V, La Torre G, Montalto M, Cammarota G, Ricci R, Masciana R, Forgione A, Gabrieli ML, Perotti G, et al. Increased intestinal permeability and tight junction alterations in nonalcoholic fatty liver disease. Hepatology. 2009;49:1877–1887. doi: 10.1002/hep.22848. 19291785

[161] Lassenius MI, Pietilainen KH, Kaartinen K, Pussinen PJ, Syrjanen J, Forsblom C, Porsti I, Rissanen A, Kaprio J, Mustonen J, et al. Bacterial endotoxin activity in human serum is associated with dyslipidemia, insulin resistance, obesity, and chronic inflammation. Diabetes Care. 2011;34:1809–1815. doi: 10.2337/dc10-2197. 21636801 PMC3142060

[162] Cani PD, Amar J, Iglesias MA, Poggi M, Knauf C, Bastelica D, Neyrinck AM, Fava F, Tuohy KM, Chabo C, et al. Metabolic endotoxemia initiates obesity and insulin resistance. Diabetes. 2007;56:1761–1772. doi: 10.2337/db06-1491. 17456850

[163] Camargo A, Jimenez-Lucena R, Alcala-Diaz JF, Rangel-Zuniga OA, Garcia-Carpintero S, Lopez-Moreno J, Blanco-Rojo R, Delgado-Lista J, Perez-Martinez P, van Ommen B, et al. Postprandial endotoxemia May influence the development of type 2 diabetes mellitus: from the CORDIOPREV study. Clin Nutr. 2019;38:529–538. doi: 10.1016/j.clnu.2018.03.016. 29685478

[164] Mohammad S, Thiemermann C. Role of metabolic endotoxemia in systemic inflammation and potential interventions. Front Immunol. 2020;11:594150. doi: 10.3389/fimmu.2020.594150. 33505393 PMC7829348

[165] Creely SJ, McTernan PG, Kusminski CM, Fisher M, Da Silva NF, Khanolkar M, Evans M, Harte AL, Kumar S. Lipopolysaccharide activates an innate immune system response in human adipose tissue in obesity and type 2 diabetes. Am J Physiol Endocrinol Metab. 2007;292:E740–7. doi: 10.1152/ajpendo.00302.2006. 17090751

[166] Pussinen PJ, Havulinna AS, Lehto M, Sundvall J, Salomaa V. Endotoxemia is associated with an increased risk of incident diabetes. Diabetes Care. 2011;34:392–397. doi: 10.2337/dc10-1676. 21270197 PMC3024355

[167] Pendyala S, Walker JM, Holt PR. A high-fat diet is associated with endotoxemia that originates from the gut. Gastroenterology. 2012;142:1100–1101 e2. doi: 10.1053/j.gastro.2012.01.034. 22326433 PMC3978718

[168] Wigg AJ, Roberts-Thomson IC, Dymock RB, McCarthy PJ, Grose RH, Cummins AG. The role of small intestinal bacterial overgrowth, intestinal permeability, endotoxaemia, and tumour necrosis factor alpha in the pathogenesis of non-alcoholic steatohepatitis. Gut. 2001;48:206–211. doi: 10.1136/gut.48.2.206. 11156641 PMC1728215

[169] Pang J, Xu W, Zhang X, Wong GL, Chan AW, Chan HY, Tse CH, Shu SS, Choi PC, Chan HL, et al. Significant positive association of endotoxemia with histological severity in 237 patients with non-alcoholic fatty liver disease. Aliment Pharmacol Ther. 2017;46:175–182. doi: 10.1111/apt.14119. 28464257

[170] Soppert J, Brandt EF, Heussen NM, Barzakova E, Blank LM, Kuepfer L, Hornef MW, Trebicka J, Jankowski J, Berres ML, et al. Blood endotoxin levels as biomarker of nonalcoholic fatty liver disease: a systematic review and meta-analysis. Clin Gastroenterol Hepatol. 2023;21:2746–2758. doi: 10.1016/j.cgh.2022.11.030. 36470528

[171] Guo H, Diao N, Yuan R, Chen K, Geng S, Li M, Li L. Subclinical-dose endotoxin sustains low-grade inflammation and exacerbates steatohepatitis in high-fat diet-fed mice. J Immunol. 2016;196:2300–2308. doi: 10.4049/jimmunol.1500130. 26810228 PMC4761480

[172] Cani PD, Bibiloni R, Knauf C, Waget A, Neyrinck AM, Delzenne NM, Burcelin R. Changes in gut microbiota control metabolic endotoxemia-induced inflammation in high-fat diet-induced obesity and diabetes in mice. Diabetes. 2008;57:1470–1481. doi: 10.2337/db07-1403. 18305141

[173] Pena-Duran E, Garcia-Galindo JJ, Lopez-Murillo LD, Huerta-Huerta A, Balleza-Alejandri LR, Beltran-Ramirez A, Anaya-Ambriz EJ, Suarez-Rico DO. Microbiota and inflammatory markers: a review of their interplay, clinical implications, and metabolic disorders. Int J Mol Sci. 2025;26:1773. doi: 10.3390/ijms26041773. 40004236 PMC11854938

[174] Luria A, Bettaieb A, Xi Y, Shieh GJ, Liu HC, Inoue H, Tsai HJ, Imig JD, Haj FG, Hammock BD. Soluble epoxide hydrolase deficiency alters pancreatic islet size and improves glucose homeostasis in a model of insulin resistance. Proc Natl Acad Sci U S A. 2011;108:9038–9043. doi: 10.1073/pnas.1103482108. 21571638 PMC3107315

[175] Shiratori H, Oguchi H, Isobe Y, Han KH, Sen A, Yakebe K, Takahashi D, Fukushima M, Arita M, Hase K. Gut microbiota-derived lipid metabolites facilitate regulatory T cell differentiation. Sci Rep. 2023;13:8903. doi: 10.1038/s41598-023-35097-5. 37264064 PMC10235104

[176] Grimes D, Watson D. Epoxyeicosatrienoic acids protect pancreatic beta cells against pro-inflammatory cytokine toxicity. Biochem Biophys Res Commun. 2019;520:231–236. doi: 10.1016/j.bbrc.2019.09.124. 31590920

[177] Xu X, Zhao CX, Wang L, Tu L, Fang X, Zheng C, Edin ML, Zeldin DC, Wang DW. Increased CYP2J3 expression reduces insulin resistance in fructose-treated rats and db/db mice. Diabetes. 2010;59:997–1005. doi: 10.2337/db09-1241. 20068141 PMC2844847

[178] Lin AZ, Fu X, Jiang Q, Zhou X, Hwang SH, Yin HH, Ni KD, Pan QJ, He X, Zhang LT, et al. Metabolomics reveals soluble epoxide hydrolase as a therapeutic target for high-sucrose diet-mediated gut barrier dysfunction. Proc Natl Acad Sci U S A. 2024;121:e2409841121. doi: 10.1073/pnas.2409841121. 39556751 PMC11621843

[179] Kalveram L, Schunck WH, Rothe M, Rudolph B, Loddenkemper C, Holzhutter HG, Henning S, Bufler P, Schulz M, Meierhofer D, et al. Regulation of the cytochrome P450 epoxyeicosanoid pathway is associated with distinct histologic features in pediatric non-alcoholic fatty liver disease. Prostaglandins Leukot Essent Fatty Acids. 2021;164:102229. doi: 10.1016/j.plefa.2020.102229. 33388475

[180] Liu Y, Dang H, Li D, Pang W, Hammock BD, Zhu Y. Inhibition of soluble epoxide hydrolase attenuates high-fat-diet-induced hepatic steatosis by reduced systemic inflammatory status in mice. PLoS One. 2012;7:e39165. doi: 10.1371/journal.pone.0039165. 22720061 PMC3375303

[181] Wells MA, Vendrov KC, Edin ML, Ferslew BC, Zha W, Nguyen BK, Church RJ, Lih FB, DeGraff LM, Brouwer KL, et al. Characterization of the cytochrome P450 epoxyeicosanoid pathway in non-alcoholic steatohepatitis. Prostaglandins Other Lipid Mediat. 2016;125:19–29. doi: 10.1016/j.prostaglandins.2016.07.002. 27401401 PMC5035202

[182] Warner DR, Warner JB, Abdelfadil Y, Hardesty JE, Treves R, Lei C, Hanford HE, McClain CJ, Kirpich IA. Effects of soluble epoxide hydrolase inhibition on liver injury and gut microbiota in mice chronically fed ethanol. Alcohol Clin Exp Res (Hoboken). 2025;49:1730–1743. doi: 10.1111/acer.70109. 40611388 PMC12266649

[183] Bettaieb A, Nagata N, AbouBechara D, Chahed S, Morisseau C, Hammock BD, Haj FG. Soluble epoxide hydrolase deficiency or inhibition attenuates diet-induced endoplasmic reticulum stress in liver and adipose tissue. J Biol Chem. 2013;288:14189–14199. doi: 10.1074/jbc.M113.458414. 23576437 PMC3656275

[184] Harris TR, Bettaieb A, Kodani S, Dong H, Myers R, Chiamvimonvat N, Haj FG, Hammock BD. Inhibition of soluble epoxide hydrolase attenuates hepatic fibrosis and endoplasmic reticulum stress induced by carbon tetrachloride in mice. Toxicol Appl Pharmacol. 2015;286:102–111. doi: 10.1016/j.taap.2015.03.022. 25827057 PMC4458210

[185] Zhou D, Pan Q, Shen F, Cao HX, Ding WJ, Chen YW, Fan JG. Total fecal microbiota transplantation alleviates high-fat diet-induced steatohepatitis in mice via beneficial regulation of gut microbiota. Sci Rep. 2017;7:1529. doi: 10.1038/s41598-017-01751-y. 28484247 PMC5431549

[186] Suk KT, Koh H. New perspective on fecal microbiota transplantation in liver diseases. J Gastroenterol Hepatol. 2022;37:24–33. doi: 10.1111/jgh.15729. 34734433

[187] Abenavoli L, Maurizi V, Rinninella E, Tack J, Di Berardino A, Santori P, Rasetti C, Procopio AC, Boccuto L, Scarpellini E. Fecal microbiota transplantation in NAFLD treatment. Medicina (Kaunas). 2022;58:1559. doi: 10.3390/medicina58111559. 36363516 PMC9695159

[188] Xue L, Deng Z, Luo W, He X, Chen Y. Effect of fecal microbiota transplantation on non-alcoholic fatty liver disease: a randomized clinical trial. Front Cell Infect Microbiol. 2022;12:759306. doi: 10.3389/fcimb.2022.759306. 35860380 PMC9289257

[189] Yang Y, Yan J, Li S, Liu M, Han R, Wang Y, Wang Z, Wang D. Efficacy of fecal microbiota transplantation in type 2 diabetes mellitus: a systematic review and meta-analysis. Endocrine. 2024;84:48–62. doi: 10.1007/s12020-023-03606-1. 38001323

[190] Wang H, Li S, Zhang L, Zhang N. The role of fecal microbiota transplantation in type 2 diabetes mellitus treatment. Front Endocrinol (Lausanne). 2024;15:1469165. doi: 10.3389/fendo.2024.1469165. 39735647 PMC11671274

[191] Wu Z, Zhang B, Chen F, Xia R, Zhu D, Chen B, Lin A, Zheng C, Hou D, Li X, et al. Fecal microbiota transplantation reverses insulin resistance in type 2 diabetes: a randomized, controlled, prospective study. Front Cell Infect Microbiol. 2022;12:1089991. doi: 10.3389/fcimb.2022.1089991. 36704100 PMC9872724

[192] Ng SC, Xu Z, Mak JWY, Yang K, Liu Q, Zuo T, Tang W, Lau L, Lui RN, Wong SH, et al. Microbiota engraftment after faecal microbiota transplantation in obese subjects with type 2 diabetes: a 24-week, double-blind, randomised controlled trial. Gut. 2022;71:716–723. doi: 10.1136/gutjnl-2020-323617. 33785557

[193] Chen L, Guo L, Feng S, Wang C, Cui Z, Wang S, Lu Q, Chang H, Hang B, Snijders AM, et al. Fecal microbiota transplantation ameliorates type 2 diabetes via metabolic remodeling of the gut microbiota in db/db mice. BMJ Open Diabetes Res Care. 2023;11:e003282. doi: 10.1136/bmjdrc-2022-003282. PMC10230930 37253485

[194] Pingitore A, Gonzalez-Abuin N, Ruz-Maldonado I, Huang GC, Frost G, Persaud SJ. Short chain fatty acids stimulate insulin secretion and reduce apoptosis in mouse and human islets in vitro: role of free fatty acid receptor 2. Diabetes Obes Metab. 2019;21:330–339. doi: 10.1111/dom.13529. 30203438

[195] Chambers ES, Viardot A, Psichas A, Morrison DJ, Murphy KG, Zac-Varghese SE, MacDougall K, Preston T, Tedford C, Finlayson GS, et al. Effects of targeted delivery of propionate to the human colon on appetite regulation, body weight maintenance and adiposity in overweight adults. Gut. 2015;64:1744–1754. doi: 10.1136/gutjnl-2014-307913. 25500202 PMC4680171

[196] Wang N, Guo DY, Tian X, Lin HP, Li YP, Chen SJ, Fu YC, Xu WC, Wei CJ. Niacin receptor GPR109A inhibits insulin secretion and is down-regulated in type 2 diabetic islet beta-cells. Gen Comp Endocrinol. 2016;237:98–108. doi: 10.1016/j.ygcen.2016.08.011. 27570060

[197] Ye J, Lv L, Wu W, Li Y, Shi D, Fang D, Guo F, Jiang H, Yan R, Ye W, et al. Butyrate protects mice against methionine-choline-deficient diet-induced non-alcoholic steatohepatitis by improving gut barrier function, attenuating inflammation and reducing endotoxin levels. Front Microbiol. 2018;9:1967. doi: 10.3389/fmicb.2018.01967. 30186272 PMC6111843

[198] Deng M, Qu F, Chen L, Liu C, Zhang M, Ren F, Guo H, Zhang H, Ge S, Wu C, et al. SCFAs alleviated steatosis and inflammation in mice with NASH induced by MCD. J Endocrinol. 2020;245:425–437. doi: 10.1530/JOE-20-0018. 32302970

[199] Zheng M, Yang X, Wu Q, Gong Y, Pang N, Ge X, Nagaratnam N, Jiang P, Zhou M, Hu T, et al. Butyrate attenuates hepatic steatosis induced by a high-fat and fiber-deficient diet via the hepatic GPR41/43-CaMKII/HDAC1-CREB pathway. Mol Nutr Food Res. 2023;67:e2200597. doi: 10.1002/mnfr.202200597. 36382553 PMC10078002

[200] Weitkunat K, Schumann S, Nickel D, Kappo KA, Petzke KJ, Kipp AP, Blaut M, Klaus S. Importance of propionate for the repression of hepatic lipogenesis and improvement of insulin sensitivity in high-fat diet-induced obesity. Mol Nutr Food Res. 2016;60:2611–2621. doi: 10.1002/mnfr.201600305. 27467905 PMC5215627

[201] Norris PC, Skulas-Ray AC, Riley I, Richter CK, Kris-Etherton PM, Jensen GL, Serhan CN, Maddipati KR. Identification of specialized pro-resolving mediator clusters from healthy adults after intravenous low-dose endotoxin and omega-3 supplementation: a methodological validation. Sci Rep. 2018;8:18050. doi: 10.1038/s41598-018-36679-4. 30575798 PMC6303400

[202] Xia J, Yin S, Yu J, Wang J, Jin X, Wang Y, Liu H, Sun G. Improvement in glycolipid metabolism parameters after supplementing fish oil-derived Omega-3 fatty acids is associated with gut microbiota and lipid metabolites in type 2 diabetes mellitus. Nutrients. 2024;16:3755. doi: 10.3390/nu16213755. 39519588 PMC11547733

[203] Costantini L, Molinari R, Farinon B, Merendino N. Impact of Omega-3 fatty acids on the gut microbiota. Int J Mol Sci. 2017;18:2645. doi: 10.3390/ijms18122645. 29215589 PMC5751248

[204] Stankovic MN, Mladenovic DR, Duricic I, Sobajic SS, Timic J, Jorgacevic B, Aleksic V, Vucevic DB, Jesic-Vukicevic R, Radosavljevic TS. Time-dependent changes and association between liver free fatty acids, serum lipid profile and histological features in mice model of nonalcoholic fatty liver disease. Arch Med Res. 2014;45:116–124. doi: 10.1016/j.arcmed.2013.12.010. 24480733

[205] Maciejewska-Markiewicz D, Drozd A, Palma J, Ryterska K, Hawrylkowicz V, Zaleska P, Wunsh E, Kozlowska-Petriczko K, Stachowska E. Fatty acids and eicosanoids change during high-fiber diet in NAFLD patients-randomized control trials (RCT). Nutrients. 2022;14:4310. doi: 10.3390/nu14204310. 36296994 PMC9608825

[206] Fenske RJ, Weeks AM, Daniels M, Nall R, Pabich S, Brill AL, Peter DC, Punt M, Cox ED, Davis DB, et al. Plasma prostaglandin E(2) metabolite levels predict type 2 diabetes status and one-year therapeutic response independent of clinical markers of inflammation. Metabolites. 2022;12:1234. doi: 10.3390/metabo12121234. 36557272 PMC9783643

[207] Tian F, Zhang YJ, Li Y, Xie Y. Celecoxib ameliorates non-alcoholic steatohepatitis in type 2 diabetic rats via suppression of the non-canonical wnt signaling pathway expression. PLoS One. 2014;9:e83819. doi: 10.1371/journal.pone.0083819. 24404139 PMC3880264

[208] Henkel J, Coleman CD, Schraplau A, Johrens K, Weiss TS, Jonas W, Schurmann A, Puschel GP. Augmented liver inflammation in a microsomal prostaglandin E synthase 1 (mPGES-1)-deficient diet-induced mouse NASH model. Sci Rep. 2018;8:16127. doi: 10.1038/s41598-018-34633-y. 30382148 PMC6208405

[209] Bosma KJ, Andrei SR, Katz LS, Smith AA, Dunn JC, Ricciardi VF, Ramirez MA, Baumel-Alterzon S, Pace WA, Carroll DT, et al. Pharmacological blockade of the EP3 prostaglandin E(2) receptor in the setting of type 2 diabetes enhances beta-cell proliferation and identity and relieves oxidative damage. Mol Metab. 2021;54:101347. doi: 10.1016/j.molmet.2021.101347. 34626853 PMC8529552

[210] Schaid MD, Wisinski JA, Kimple ME. The EP3 Receptor/G(z) signaling axis as a therapeutic target for diabetes and cardiovascular disease. AAPS J. 2017;19:1276–1283. doi: 10.1208/s12248-017-0097-1. 28584908 PMC7934137

[211] Scher JU, Sczesnak A, Longman RS, Segata N, Ubeda C, Bielski C, Rostron T, Cerundolo V, Pamer EG, Abramson SB, et al. Expansion of intestinal prevotella copri correlates with enhanced susceptibility to arthritis. Elife. 2013;2:e01202. doi: 10.7554/eLife.01202. 24192039 PMC3816614

[212] Chen J, Wright K, Davis JM, Jeraldo P, Marietta EV, Murray J, Nelson H, Matteson EL, Taneja V. An expansion of rare lineage intestinal microbes characterizes rheumatoid arthritis. Genome Med. 2016;8:43. doi: 10.1186/s13073-016-0299-7. 27102666 PMC4840970

[213] Thompson KN, Bonham KS, Ilott NE, Britton GJ, Colmenero P, Bullers SJ, McIver LJ, Ma S, Nguyen LH, Filer A, et al. Inflammatory Arthritis Microbiome Consortium investigators g Iamc. Alterations in the gut microbiome implicate key taxa and metabolic pathways across inflammatory arthritis phenotypes. Sci Transl Med. 2023;15 eabn4722. doi: 10.1126/scitranslmed.abn4722. 37494472

[214] Zhang X, Zhang D, Jia H, Feng Q, Wang D, Liang D, Wu X, Li J, Tang L, Li Y, et al. The oral and gut microbiomes are perturbed in rheumatoid arthritis and partly normalized after treatment. Nat Med. 2015;21:895–905. doi: 10.1038/nm.3914. 26214836

[215] Breban M, Tap J, Leboime A, Said-Nahal R, Langella P, Chiocchia G, Furet JP, Sokol H. Faecal microbiota study reveals specific dysbiosis in spondyloarthritis. Ann Rheum Dis. 2017;76:1614–1622. doi: 10.1136/annrheumdis-2016-211064. 28606969

[216] Hong M, Li Z, Liu H, Zheng S, Zhang F, Zhu J, Shi H, Ye H, Chou Z, Gao L, et al. Fusobacterium nucleatum aggravates rheumatoid arthritis through FadA-containing outer membrane vesicles. Cell Host Microbe. 2023;31:798–810 e7. doi: 10.1016/j.chom.2023.03.018. 37054714

[217] Chriswell ME, Lefferts AR, Clay MR, Hsu AR, Seifert J, Feser ML, Rims C, Bloom MS, Bemis EA, Liu S, et al. Clonal IgA and IgG autoantibodies from individuals at risk for rheumatoid arthritis identify an arthritogenic strain of. Subdoligranulum. Sci Transl Med. 2022;14:eabn5166. doi: 10.1126/scitranslmed.abn5166. 36288282 PMC9804515

[218] Yu D, Du J, Pu X, Zheng L, Chen S, Wang N, Li J, Chen S, Pan S, Shen B. The gut microbiome and metabolites are altered and interrelated in patients with rheumatoid arthritis. Front Cell Infect Microbiol. 2021;11:763507. doi: 10.3389/fcimb.2021.763507. 35145919 PMC8821809

[219] Berland M, Meslier V, Berreira Ibraim S, Le Chatelier E, Pons N, Maziers N, Thirion F, Gauthier F, Plaza Onate F, Furet JP, et al. Both disease activity and HLA-B27 status are associated with gut microbiome dysbiosis in spondyloarthritis patients. Arthritis Rheumatol. 2023;75:41–52. doi: 10.1002/art.42289. 35818337 PMC10099252

[220] Tito RY, Cypers H, Joossens M, Varkas G, Van Praet L, Glorieus E, Van den Bosch F, De Vos M, Raes J, Elewaut D. Brief report: dialister as a microbial marker of disease activity in spondyloarthritis. Arthritis Rheumatol. 2017;69:114–121. doi: 10.1002/art.39802. 27390077

[221] Stoll ML, Sawhney H, Wells PM, Sternes PR, Reveille JD, Morrow CD, Steves CJ, Brown MA, Gensler LS. The faecal microbiota is distinct in HLA-B27+ ankylosing spondylitis patients versus HLA-B27+ healthy controls. Clin Exp Rheumatol. 2023;41:1096–1104. doi: 10.55563/clinexprheumatol/nlsj0o. 36441657

[222] Scher JU, Ubeda C, Artacho A, Attur M, Isaac S, Reddy SM, Marmon S, Neimann A, Brusca S, Patel T, et al. Decreased bacterial diversity characterizes the altered gut microbiota in patients with psoriatic arthritis, resembling dysbiosis in inflammatory bowel disease. Arthritis Rheumatol. 2015;67:128–139. doi: 10.1002/art.38892. 25319745 PMC4280348

[223] Min HK, Na HS, Jhun J, Lee SY, Choi SS, Park GE, Lee JS, Um IG, Lee SY, Seo H, et al. Identification of gut dysbiosis in axial spondyloarthritis patients and improvement of experimental ankylosing spondyloarthritis by microbiome-derived butyrate with immune-modulating function. Front Immunol. 2023;14:1096565. doi: 10.3389/fimmu.2023.1096565. 37143677 PMC10152063

[224] Abebaw D, Akelew Y, Adugna A, Tegegne BA, Teffera ZH, Belayneh M, Fenta A, Selabat B, Kindie Y, Baylie T, et al. Immunomodulatory properties of the gut microbiome: diagnostic and therapeutic potential for rheumatoid arthritis. Clin Exp Med. 2025;25:226. doi: 10.1007/s10238-025-01777-x. 40591032 PMC12214023

[225] He J, Chu Y, Li J, Meng Q, Liu Y, Jin J, Wang Y, Wang J, Huang B, Shi L, et al. Intestinal butyrate-metabolizing species contribute to autoantibody production and bone erosion in rheumatoid arthritis. Sci Adv. 2022;8:eabm1511. doi: 10.1126/sciadv.abm1511. 35148177 PMC11093108

[226] Paine A, Brookes PS, Bhattacharya S, Li D, De La Luz Garcia-Hernandez M, Tausk F, Ritchlin C. Dysregulation of bile acids, lipids, and nucleotides in psoriatic arthritis revealed by unbiased profiling of serum metabolites. Arthritis Rheumatol. 2023;75:53–63. doi: 10.1002/art.42288. 35818333 PMC9797425

[227] Martinsson K, Durholz K, Schett G, Zaiss MM, Kastbom A. Higher serum levels of short-chain fatty acids are associated with non-progression to arthritis in individuals at increased risk of RA. Ann Rheum Dis. 2022;81:445–447. doi: 10.1136/annrheumdis-2021-221386. 34819270 PMC8862054

[228] Leipe J, Grunke M, Dechant C, Reindl C, Kerzendorf U, Schulze-Koops H, Skapenko A. Role of Th17 cells in human autoimmune arthritis. Arthritis Rheum. 2010;62:2876–2885. doi: 10.1002/art.27622. 20583102

[229] Ivanov II, Atarashi K, Manel N, Brodie EL, Shima T, Karaoz U, Wei D, Goldfarb KC, Santee CA, Lynch SV, et al. Induction of intestinal Th17 cells by segmented filamentous bacteria. Cell. 2009;139:485–498. doi: 10.1016/j.cell.2009.09.033. 19836068 PMC2796826

[230] Goto Y, Panea C, Nakato G, Cebula A, Lee C, Diez MG, Laufer TM, Ignatowicz L, Ivanov II. Segmented filamentous bacteria antigens presented by intestinal dendritic cells drive mucosal Th17 cell differentiation. Immunity. 2014;40:594–607. doi: 10.1016/j.immuni.2014.03.005. 24684957 PMC4084624

[231] Wu HJ, Ivanov II, Darce J, Hattori K, Shima T, Umesaki Y, Littman DR, Benoist C, Mathis D. Gut-residing segmented filamentous bacteria drive autoimmune arthritis via T helper 17 cells. Immunity. 2010;32:815–827. doi: 10.1016/j.immuni.2010.06.001. 20620945 PMC2904693

[232] Viladomiu M, Kivolowitz C, Abdulhamid A, Dogan B, Victorio D, Castellanos JG, Woo V, Teng F, Tran NL, Sczesnak A, et al. Coli enriched in Crohn's disease spondyloarthritis promote T(H)17-dependent inflammation. Sci Transl Med. 2017;9. doi: 10.1126/scitranslmed.aaf9655. PMC6159892 28179509

[233] Asquith M, Davin S, Stauffer P, Michell C, Janowitz C, Lin P, Ensign-Lewis J, Kinchen JM, Koop DR, Rosenbaum JT. Intestinal metabolites are profoundly altered in the context of HLA-B27 expression and functionally modulate disease in a rat model of spondyloarthritis. Arthritis Rheumatol. 2017;69:1984–1995. doi: 10.1002/art.40183. 28622455 PMC5623151

[234] Rosser EC, Piper CJM, Matei DE, Blair PA, Rendeiro AF, Orford M, Alber DG, Krausgruber T, Catalan D, Klein N, et al. Microbiota-derived metabolites suppress arthritis by amplifying aryl-hydrocarbon receptor activation in regulatory B cells. Cell Metab. 2020;31:837–851 e10. doi: 10.1016/j.cmet.2020.03.003. 32213346 PMC7156916

[235] Lucas S, Omata Y, Hofmann J, Bottcher M, Iljazovic A, Sarter K, Albrecht O, Schulz O, Krishnacoumar B, Kronke G, et al. Short-chain fatty acids regulate systemic bone mass and protect from pathological bone loss. Nat Commun. 2018;9:55. doi: 10.1038/s41467-017-02490-4. 29302038 PMC5754356

[236] Durholz K, Hofmann J, Iljazovic A, Hager J, Lucas S, Sarter K, Strowig T, Bang H, Rech J, Schett G, et al. Dietary short-term fiber interventions in arthritis patients increase systemic SCFA levels and regulate inflammation. Nutrients. 2020;12:3207. doi: 10.3390/nu12103207. 33092271 PMC7589100

[237] Hager J, Bang H, Hagen M, Frech M, Trager P, Sokolova MV, Steffen U, Tascilar K, Sarter K, Schett G, et al. The role of dietary fiber in rheumatoid arthritis patients: a feasibility study. Nutrients. 2019;11:2392. doi: 10.3390/nu11102392. 31591345 PMC6836071

[238] Inoue H, Takamori M, Shimoyama Y, Ishibashi H, Yamamoto S, Koshihara Y. Regulation by PGE2 of the production of interleukin-6, macrophage colony stimulating factor, and vascular endothelial growth factor in human synovial fibroblasts. Br J Pharmacol. 2002;136:287–295. doi: 10.1038/sj.bjp.0704705. 12010778 PMC1573344

[239] Honda T, Segi-Nishida E, Miyachi Y, Narumiya S. Prostacyclin-IP signaling and prostaglandin E2-EP2/EP4 signaling both mediate joint inflammation in mouse collagen-induced arthritis. J Exp Med. 2006;203:325–335. doi: 10.1084/jem.20051310. 16446378 PMC2118213

[240] Mauro D, Srinath A, Guggino G, Nicolaidou V, Raimondo S, Ellis JJ, Whyte J, Nicoletti MM, Romano M, Kenna TJ, et al. Prostaglandin E2/EP4 axis is upregulated in spondyloarthritis and contributes to radiographic progression. Clin Immunol. 2023;251:109332. doi: 10.1016/j.clim.2023.109332. 37075950

[241] Wei X, Zhang X, Flick LM, Drissi H, Schwarz EM, O'Keefe RJ. Titanium particles stimulate COX-2 expression in synovial fibroblasts through an oxidative stress-induced, calpain-dependent, NF-kappaB pathway. Am J Physiol Cell Physiol. 2009;297:C310–20. doi: 10.1152/ajpcell.00597.2008. 19494233 PMC2724098

[242] Tsutsumi R, Xie C, Wei X, Zhang M, Zhang X, Flick LM, Schwarz EM, O'Keefe RJ. PGE2 signaling through the EP4 receptor on fibroblasts upregulates RANKL and stimulates osteolysis. J Bone Miner Res. 2009;24:1753–1762. doi: 10.1359/jbmr.090412. 19419302 PMC2743284

[243] Ono K, Akatsu T, Kugai N, Pilbeam CC, Raisz LG. The effect of deletion of cyclooxygenase-2, prostaglandin receptor EP2, or EP4 in bone marrow cells on osteoclasts induced by mouse mammary cancer cell lines. Bone. 2003;33:798–804. doi: 10.1016/s8756-3282(03)00264-3. 14623055

[244] Suzawa T, Miyaura C, Inada M, Maruyama T, Sugimoto Y, Ushikubi F, Ichikawa A, Narumiya S, Suda T. The role of prostaglandin E receptor subtypes (EP1, EP2, EP3, and EP4) in bone resorption: an analysis using specific agonists for the respective EPs. Endocrinology. 2000;141:1554–1559. doi: 10.1210/endo.141.4.7405. 10746663

[245] Miyaura C, Inada M, Suzawa T, Sugimoto Y, Ushikubi F, Ichikawa A, Narumiya S, Suda T. Impaired bone resorption to prostaglandin E2 in prostaglandin E receptor EP4-knockout mice. J Biol Chem. 2000;275:19819–19823. doi: 10.1074/jbc.M002079200. 10749873

[246] Emery P, Zeidler H, Kvien TK, Guslandi M, Naudin R, Stead H, Verburg KM, Isakson PC, Hubbard RC, Geis GS. Celecoxib versus diclofenac in long-term management of rheumatoid arthritis: randomised double-blind comparison. Lancet. 1999;354:2106–2111. doi: 10.1016/S0140-6736(99)02332-6. 10609815

[247] Sieper J, Klopsch T, Richter M, Kapelle A, Rudwaleit M, Schwank S, Regourd E, May M. Comparison of two different dosages of celecoxib with diclofenac for the treatment of active ankylosing spondylitis: results of a 12-week randomised, double-blind, controlled study. Ann Rheum Dis. 2008;67:323–329. doi: 10.1136/ard.2007.075309. 17616556

[248] Chen Q, Muramoto K, Masaaki N, Ding Y, Yang H, Mackey M, Li W, Inoue Y, Ackermann K, Shirota H, et al. A novel antagonist of the prostaglandin E(2) EP(4) receptor inhibits Th1 differentiation and Th17 expansion and is orally active in arthritis models. Br J Pharmacol. 2010;160:292–310. doi: 10.1111/j.1476-5381.2010.00647.x. 20423341 PMC2874852

[249] Oka H, Miyauchi M, Furusho H, Nishihara T, Takata T. Oral administration of prostaglandin E(2)-specific receptor 4 antagonist inhibits lipopolysaccharide-induced osteoclastogenesis in rat periodontal tissue. J Periodontol. 2012;83:506–513. doi: 10.1902/jop.2011.110301. 21910594

[250] Gheorghe KR, Korotkova M, Catrina AI, Backman L, af Klint E, Claesson HE, Radmark O, Jakobsson PJ. Expression of 5-lipoxygenase and 15-lipoxygenase in rheumatoid arthritis synovium and effects of intraarticular glucocorticoids. Arthritis Res Ther. 2009;11:R83. doi: 10.1186/ar2717. 19497113 PMC2714134

[251] Wojcik P, Biernacki M, Wronski A, Luczaj W, Waeg G, Zarkovic N, Skrzydlewska E. Altered lipid metabolism in blood mononuclear cells of psoriatic patients indicates differential changes in psoriasis vulgaris and psoriatic arthritis. Int J Mol Sci. 2019;20:4249. doi: 10.3390/ijms20174249. 31480263 PMC6747546

[252] Xu S, Lu H, Lin J, Chen Z, Jiang D. Regulation of TNFalpha and IL1beta in rheumatoid arthritis synovial fibroblasts by leukotriene B4. Rheumatol Int. 2010;30(9):1183–1189. doi: 10.1007/s00296-009-1125-y. 19809821

[253] Bi D, Bi D, Zhong M, Zhang H, Jin S, Ma S, Luo H. Effects of leukotriene B4 on interleukin-32, interferon-gamma and chemokines in rats with rheumatoid arthritis. Exp Ther Med. 2017;14:2925–2930. doi: 10.3892/etm.2017.4845. 28912850 PMC5585718

[254] Dixit N, Wu DJ, Belgacem YH, Borodinsky LN, Gershwin ME, Adamopoulos IE. Leukotriene B4 activates intracellular calcium and augments human osteoclastogenesis. Arthritis Res Ther. 2014;16:496. doi: 10.1186/s13075-014-0496-y. 25443625 PMC4276054

[255] Dahlke P, Peltner LK, Jordan PM, Werz O. Differential impact of 5-lipoxygenase-activating protein antagonists on the biosynthesis of leukotrienes and of specialized pro-resolving mediators. Front Pharmacol. 2023;14:1219160. doi: 10.3389/fphar.2023.1219160. 37680719 PMC10481534

[256] Cerchia C, Kufner L, Werz O, Lavecchia A. Identification of selective 5-LOX and FLAP inhibitors as novel anti-inflammatory agents by ligand-based virtual screening. Eur J Med Chem. 2024;263:115932. doi: 10.1016/j.ejmech.2023.115932. 37976708

[257] Diaz-Gonzalez F, Alten RH, Bensen WG, Brown JP, Sibley JT, Dougados M, Bombardieri S, Durez P, Ortiz P, de-Miquel G, et al. Clinical trial of a leucotriene B4 receptor antagonist, BIIL 284, in patients with rheumatoid arthritis. Ann Rheum Dis. 2007;66:628–632. doi: 10.1136/ard.2006.062554. 17170051 PMC1954613

[258] Alten R, Gromnica-Ihle E, Pohl C, Emmerich J, Steffgen J, Roscher R, Sigmund R, Schmolke B, Steinmann G. Inhibition of leukotriene B4-induced CD11B/CD18 (Mac-1) expression by BIIL 284, a new long acting LTB4 receptor antagonist, in patients with rheumatoid arthritis. Ann Rheum Dis. 2004;63:170–176. doi: 10.1136/ard.2002.004499. 14722206 PMC1754875

